# Exploring the Regulation Mechanism of Xihuang Pill, *Olibanum* and β-Boswellic Acid on the Biomolecular Network of Triple-Negative Breast Cancer Based on Transcriptomics and Chemical Informatics Methodology

**DOI:** 10.3389/fphar.2020.00825

**Published:** 2020-06-11

**Authors:** Kailin Yang, Liuting Zeng, Anqi Ge, Tingting Bao, Tao Xu, Xiaobing Xie, Lifang Liu

**Affiliations:** ^1^Galactophore Department, The First Hospital of Hunan University of Chinese Medicine, Changsha, China; ^2^Department of Cardiac Surgery, Beijing Anzhen Hospital, Capital Medical University, Beijing, China; ^3^Graduate College, Capital Medical University, Beijing, China; ^4^Graduate College, Chinese Academy of Medical Sciences & Peking Union Medical College, Beijing, China; ^5^School of Medicine, Hunan University of Chinese Medicine, Changsha, China; ^6^Department of Geratology, Xiyuan Hospital, China Academy of Chinese Medical Sciences, Beijing, China; ^7^School of Clinical Medicine (Xiyuan Hospital), Beijing University of Chinese Medicine, Beijing, China

**Keywords:** Xihuang Pill, triple negative breast cancer, transcriptomics, chemical informatics, MDA-MB-231 cell, high-throughput omics, bioinformatics, Chinese medicine

## Abstract

**Background:**

Xihuang Pill (XHP) is mainly used to treat “Ru Yan (breast cancer)”. Evidence-based medical evidence and showed that XHP improves the efficacy of chemotherapy and reduced chemotherapy-induced toxicity in breast cancer patients. However, the mechanism of XHP against breast cancer is not clear.

**Methods:**

The effect of XHP extract on cell half-inhibitory concentration (IC50) and cell viability of MD-MB-231 cells was detected by CCK-8 method. The cell inhibition rate of MDA-MB-453 cells were detected by MTT method. Apoptosis was detected by flow cytometry, cell transfer ability was detected by Transwell method, and cell proliferation ability was detected by colony formation assay. The expression of Notch1, β-catenin and c-myc mRNA in MDA-MB-453 cells were detected by real-time fluorescence quantitative PCR. Then, chemical informatics and transcriptomics methodology was utilized to predict the potential compounds and targets of XHP, and collect triple negative breast cancer (TNBC) genes and the data of *Olibanum* and β-boswellic acid intervention MD-MB-231 cells (from GSE102891). The cytoscape software was utilized to undergo network construction and network analysis. Finally, the data from the network analysis was imported into the DAVID database for enrichment analysis of signaling pathways and biological processes.

**Results:**

The IC50 was 15.08 g/L (for MD-MB-231 cells). After interfering with MD-MB-231 cells with 15.08 g/L XHP extract for 72 h, compared with the control group, the cell viability, migration and proliferation was significantly decreased, while early apoptosis and late apoptosis were significantly increased (P < 0.01). After interfering with MDA-MB-453 cells with 6 g/L XHP extract for 72 h, compared with the control group, the cell inhibition and apoptosis rate increased, while the expression of Notch1, β-catenin and c-myc mRNA decreased. (P < 0.05). The chemical informatics and transcriptomics analysis showed that four networks were constructed and analyzed: (1) potential compounds-potential targets network of XHP; (2) XHP-TNBC PPI network; (3) DEGs PPI network of *Olibanum*-treated MD-MB 231 cells; (4) DEGs PPI network of β-boswellic acid -treated MD-MB 231 cells. Several anti-TNBC biological processes, signaling pathways, targets and so on were obtained.

**Conclusion:**

XHP may exert anti-TNBC effects through regulating biological processes, signaling pathways, targets found in this study.

## Introduction

Breast cancer is the leading cause of death among women worldwide, and the incidence rate has increased significantly in recent years, which seriously threatens women’s health (Fan et al., 2014; Desantis et al., 2017). Breast cancer is currently divided into five subtypes by coding sequence microarray technology (Prat et al., 2015; Cejalvo et al., 2018): (1) Luminal-A type; (2) Luminal-B type; (3) human epidermal growth factor receptor 2 (HER2) overexpression type; (4) base-like type; (5) normal type. Basal-like breast cancer is non-specific invasive ductal carcinoma (Jiang et al., 2019), and its ER, PR and HER2 are negative, also known as triple negative breast cancer (TNBC). At present, TNBC accounts for 10 to 17% of all breast cancers (Warner et al., 2015; Kulkarni et al., 2019). The vast majority of TNBCs are highly invasive ductal carcinomas with nuclear polymorphism, high mitotic rate, and minimal tubule formation (Kulkarni et al., 2019). TNBC is generally classified into basal cell-like type 1, basal cell-like type 2, immunoregulatory, interstitial and mesenchymal stem cell types, and luminal androgen receptor type (Shi et al., 2018; Jiang et al., 2019). TNBC is a poorly differentiated tumor with strong invasive and metastatic ability, easy to invade blood vessels, and increased recurrence rate (Jiang et al., 2019).

At present, the management and treatment measures for triple-negative breast cancer are mainly: (1) local treatment: surgery is still the first choice for local treatment (Li et al., 2019a); (2) systemic therapy: combination chemotherapy of taxane and anthracycline is currently the common choice for TNBC neoadjuvant chemotherapy, but anthracyclines have irreversible toxicity to the heart (Mangone et al., 2019); (3) targeted therapy (Jhan and Andrechek, 2017). However, due to the ineffectiveness of traditional endocrine therapy and targeted therapy, patients with TNBC sometimes have tumor metastasis very early, which seriously affects their physical and mental health (McCann et al., 2019). At present, in order to find new chemotherapy or sensitizing drugs, plants and natural products are gradually becoming the source of new TNBC drug development (Szarc Vel Szic et al., 2017). The current study found that some traditional Chinese medicine compounds and natural medicines can inhibit the proliferation, metastasis, and drug resistance of TNBC cells in a variety of ways (Baraya et al., 2017; Szarc Vel Szic et al., 2017).

Xihuang Pill (XHP) is from the *Wai Ke Quan Sheng Ji* by Wang Weide in 1740, which is mainly used to treat “Ru Yan (breast cancer)” and so on. XHP is compose of *Myrrha*, *Bovis Calculus*, *Olibanum* and *Moschus*. Systematic reviews and meta-analysis showed that XHP combined with chemotherapy significantly enhanced tumor response in breast cancer patients, improved Karnofsky performance scores and reduced chemotherapy-induced toxicity (Guo et al., 2018; Mao et al., 2019). He et al. found that XHP-containing serum increased TP53 and Bax (P < 0.05), and decreased the ratio of Bcl-2/Bax in MDA-MB-435 cells (He et al., 2018). The mechanism of anti-TNBC of XHP has been reported in many studies, such as improving the immunosuppressive state of the tumor microenvironment and reversing immune escape, thereby inhibiting tumor growth. XHP reduces the number of Treg cells by inhibiting the expression of PI3K and AKT and up-regulating the expression of AP-1 in Treg cells, thereby promoting Treg cell apoptosis (Li et al., 2018a). Other study found that the mechanism of XHP inhibition of tumors may be related to the up-regulation of gene and protein expression of MEKK1, SEK1, JNK1 and AP-1 in Treg cells in the tumor microenvironment (Su et al., 2018). Zheng et al. found that XHP can block the cell cycle of the Hs578T cell line and promote its apoptosis (Zheng et al., 2016).

**Figure 1:**
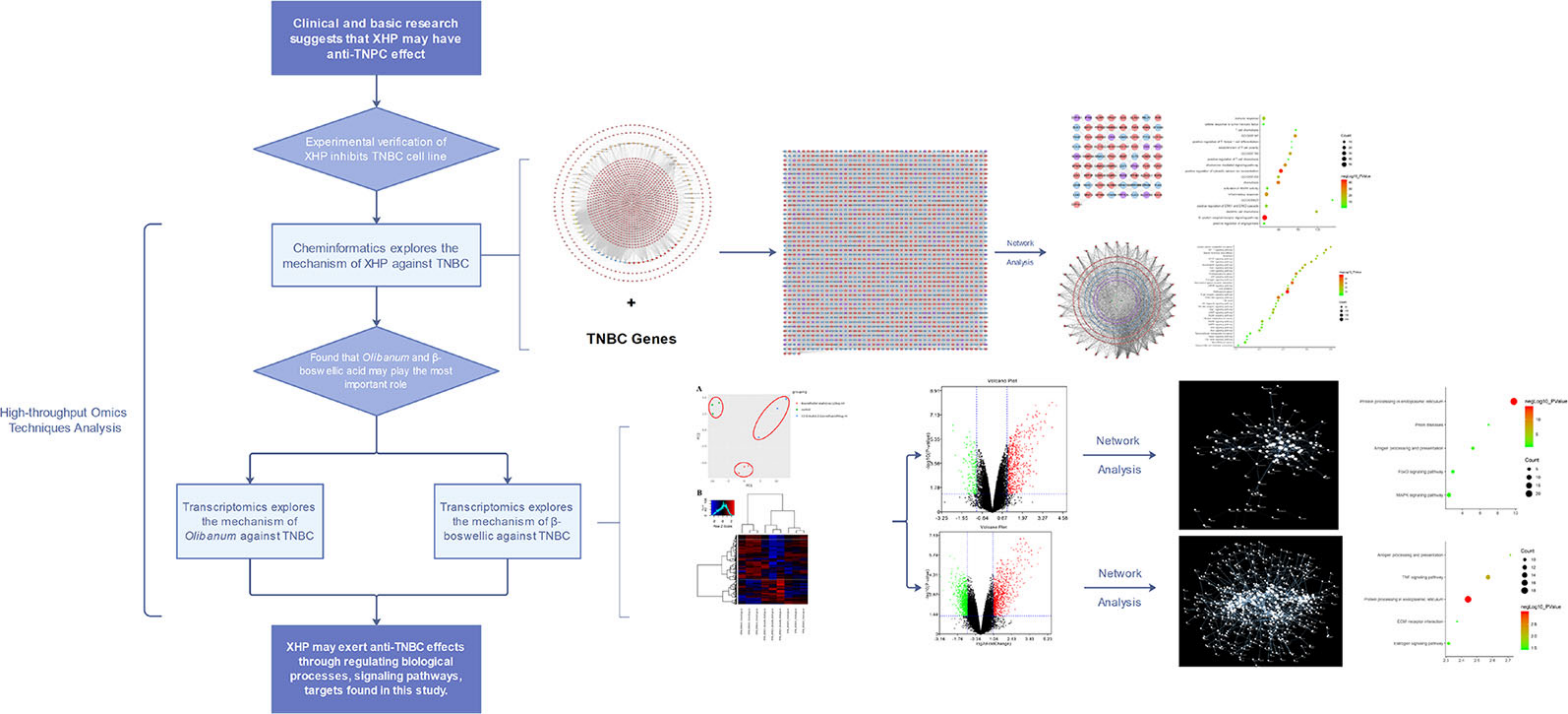
The process of this research.

Recent studies showed that the main anti-breast cancer herbs in XHP are *Myrrha* and *Olibanum*, especially *Olibanum* (Cheng et al., 2016; Hao et al., 2018). Although the above studies have described some of the mechanisms of XHP against TNBC, the mechanism remains unclear. In our previous studies, we successfully used multiple bioinformatics techniques and transcriptomics to analyze the mechanisms by which traditional Chinese Medicine interferes with different types of breast cancer (Zeng and Yang, 2017; Yang et al., 2018; Yang et al., 2019). Therefore, this study will use a multi-directional pharmacology strategy based on chemical informatics and transcriptomics to clarify the mechanisms by which XHP and *Olibanum* treat TNBC. The research process is shown in **Figure 1**.

## Material and Methods

### Experimental Material Preparation

#### Experimental Drugs

Xihuang Pill (XHP) was purchased from Tianjin Tianshili (Liaoning) Pharmaceutical Co., Ltd. (Batch number: 20140726; Specification: 0.1g * 30 bottles/box; The composition ratio of *Moschus*, *Bovis Calculus*, *Myrrha* and *Olibanum* is 15: 15: 550: 550). Reference substance: acetyl-11-keto-β-boswellic acid (batch number: 111760-201502, mass fraction >98%) was purchased from China Food and Drug Research Institute.

Preparation of XHP solution: XHP was immersed in DMEM medium pre-cooled at 4°C for 24 h (mass concentration 0.1 g/mL) in a sterile sealed container; use ultrasonic vibration to help dissolve for 2 h and continue to soak for 48 h at 4°C. The supernatant was filtered through a 0.22 μm micropore filter to obtain an XHP leaching solution. The XHP solution is stored at 4°C (or −20°C); during the experiment, it was diluted to the desired concentration with DMEM medium.

Preparation of XHP solution required for High Performance Liquid Chromatography (HPLC): 1.00 g of XHP powder was accurately weighed and placed in a 50 ml Erlenmeyer flask. Pipet 20 ml of methanol accurately, sonicate in an ice bath for 20 min, extract twice, and place at room temperature. Centrifuge at 4,000 r/min for 5 min. The supernatant was placed in a pear-shaped bottle and concentrated under reduced pressure. Reconstitute with methanol and transfer to a 25 ml volumetric flask. Finally, make up to volume with methanol and shake well.

Preparation of acetyl-11-keto-β-boswellic acid reference substance: Take an appropriate amount of acetyl-11-keto-β-boswellic acid, accurately weigh it, place it in a measuring flask, add methanol to volume, and make it to a mass concentration of 1.22 mg/mL.

#### Cell Line

Human triple-negative breast cancer (TNBC) cell line MDA-MB-231 and MDA-MB-453 were provided by the Cell Center of Xiangya School of Medicine, Central South University. While experimenting, the MD-MB-231 cells and MDA-MB-453 cells were cultured in high glucose DMEM medium containing 10% FBS in an incubator at 37°C, 5% CO2, and the medium was changed every other day. The group without XHP was the control group, and the group with XHP extract was the experimental group (XHP group), and each group had three duplicate wells.

#### Reagent and Instrument

DMEM medium and Transwell kit were purchased from Corning Inc.; Fetal bovine serun (FBS) was purchased from Ausbian Inc., Australia; CCK-8 kit and Giemsa dye solution were purchased from Sigma Inc., USA; The Annexin V-FITC/PI double-stained cell apoptosis assay kit was purchased from eBioscience Inc., USA.

Ultra-clean workbench (Sujing Antai Company), CO2 incubator (Hitachi Company). LC-20A HPLC, including SPD-M20A detector, DGU-20A quaternary pump (Shimadzu company); KQ-300DE ultrasonic cleaner (Kunshan Ultrasonic Instrument Co., Ltd.)

### HPLC Detection

Shimadzu LC-20A HPLC, Waters Symmetry C18 column (250 mm × 4.6 mm, 5 μm); injection volume 20 μl; column temperature 30°C; volume flow 0.8 ml/min; mobile phase is methanol-0.5% acetic acid aqueous solution.

The elution procedure is 0 to 10 min, 75% methanol; 10 to 45 min, 75 to 87% methanol; 45 to 75 min, 87% methanol; 75 to 85 min, 87 to 90% methanol; 85 to 115 min, 90% methanol; 115 to 120 min, 90 to 97% methanol; 120 to 150 min, 97% methanol. The detector is an evaporative light scattering detector, the drift tube temperature is 45°C; the air volume flow is 1.5 L/min, and the gain value is 2.

### Experimental Methods for MD-MB-231 Cell

#### Detection of XHP Half-Inhibitory Concentration (IC50) and Cell Viability

Some 96-well plates were seeded at 3,000 cells (100 μl) per well. Nine (9) groups were set according to the concentration of added XHP, and the concentrations of XHP were 0, 1, 2, 5, 10, 20, 50, 75 and 100 g/L. After treatment for 72 h, 10 μl of CCK-8 reagent was added 2 to 4 h before the termination of culture, and the OD value was detected by a microplate reader at 450 nm. After logarithmic processing, a scatter plot was prepared to calculate the IC50 value of XHP. Using this IC50 value as the drug concentration of XHP intervention in MD-MB-231 cells in subsequent experiments (including cell viability detection).

After treatment with XHP extract for 72 h, the culture was continued for 5 d with the control group. 10 μl of CCK-8 reagent was added 2 to 4 h before the termination of the culture, and the cell viability was measured daily for 5 days *via* the above procedure.

#### MD-MB-231 Cell Apoptosis Detection by Flow Cytometry

Some 6-well plates were seeded at a minimum of 5 × 10^5^ cells per well (2 ml) and plated for 24 h. Apoptosis detection was performed by flow cytometry according to the procedure in the instructions of Annexin V-FITC/PI double-stained cell apoptosis assay kit.

#### Transwell Detection

The cells were cultured in 24-well plates. About 100 μl of serum-free medium was added to each well of the Transwell’s inner chamber, and 600 μl of medium containing 30% FBS was added to each well of the Transwell’s outer chamber, and plated for 18 h at a cell number of 1 × 105 per well. Transfer cells, fix, stain with Giemsa stain, photograph with fluorescence microscopy, and count cells at a magnification of 200×.

#### Cell Clone Detection

Some 6-well plates were seeded at 800 cells (2 ml) per well, and continue to culture for 10 days, and change the solution once every 3 days. Cell clones were photographed under a fluorescent microscope before termination of the experiment. Fix the cells with 4% paraformaldehyde, crystal violet staining, photograph.

### Experimental Methods for MDA-MB-453 Cell

#### Determination of Cell Inhibition Rate by MTT Method

The MDA-MB-453 cells in the logarithmic growth phase were used for experiments. The cell concentration was adjusted to 1 × 10^5^ cells/mL and inoculated in 96-well culture plates; 90 μl of cell suspension was added to each well, and then 10 μl of different concentrations of XHP (0, 4, 6, 8, 10, 12, 14 g/L) were added. Six (6) duplicate wells were set for each group, and after incubating in a cell incubator at 37°C and 5% CO2 for 72 h, 20 μl MTT (5mg/mL) was added to each well. After continuing the culture for 4 h, the supernatant was discarded, and DMSO solution (DMSO) 150 μl/well was added to each well. After shaking for 10 min to fully dissolve the crystals, the OD value of each well was measured with a microplate reader at a wavelength of 490 nm.

#### MDA-MB-453 Cell Apoptosis Detection by Flow Cytometry

Apoptosis detection was performed by flow cytometry according to the procedure in the instructions of Annexin V-FITC/PI double-stained cell apoptosis assay kit.

#### The Expression of Notch1, β-catenin and c-myc mRNA Detection by Real-Time Fluorescence Quantitative PCR

The total RNA of each group of cells was extracted with Trizol according to the kit instructions. The OD260/OD280 ratio is calculated, and the ratio ≥1.8 means the purity and concentration of the RNA meet the experimental requirements. After detecting the integrity of RNA by agarose gel electrophoresis, the primers of Notch1, β-catenin, c-myc and internal reference GAPDH were added to amplify the corresponding target fragments. Finally, the real-time fluorescence quantitative PCR program was performed on the machine. The primers were designed by Primer 3.0 software and synthesized by Yuantai Bio-Technology Co., Ltd., see **Table 1**.

**Table 1:** The primers.

Gene	Primer sequence (5′->3′)	Product size (bp)	Annealing temperature (°C)
GAPDH	F: CAATGACCCCTTCATTGACC P: GACAAGCTTCCCGTTCTCAG	106	59/60
Notch1	F: ACCAATACAACCCTCTGCGG P: GGCCCTGGTAGCTCATCATC	141	59
β-catenin	F: ATGAC TCGAGCTCAGAGGGT P:ATTGCACGTGTGGCAAGTTC	99	60
C-myc	F:CGTCCTCGGATTCTCTGCTC P:GCTGCGTAGTTGTGCTGATG	186	60

### Statistical Analysis

Statistical analysis was performed using SPSS 19.0 software. The measurement data were normally distributed, expressed as mean ± standard deviation, and t-test was used for comparison between groups. The difference was statistically significant at P < 0.05.

### Chemical Informatics Methods

#### XHP Active Compounds Prediction

With the development of computer technology, Chinese medicine-related laboratories have built several large-scale Chinese medicine databases, which contain the components of commonly used Chinese medicines. Traditional Chinese Medicine Systems Pharmacology Database (TCMSP) (http://tcmspw.com/tcmsp.php) (Chen et al., 2014), Traditional Chinese Medicine Database@Taiwan (TCM@Taiwan) (http://tcm.cmu.edu.tw/zh-tw/) (Ru et al., 2014), Traditional Chinese Medicines Integrated Database (TCMID) (http://119.3.41.228:8000/tcmid/) (Lin et al., 2018) is a commonly used database. Oral bioavailability (OB), Caco-2 permeability and drug-likeness (DL) were utilized to identify the potential bioactive compounds of XHP (Walters and Murcko, 2002; Ano et al., 2004; Hu et al., 2009; Xu et al., 2012; Zeng and Yang, 2017; Yang et al., 2018; Yang et al., 2019). The compounds with OB ≥30%, Caco-2 > −0.4 and DL ≥0.18 were regard as oral absorbable compounds with biologically active (Walters and Murcko, 2002; Ano et al., 2004; Hu et al., 2009; Xu et al., 2012; Zeng and Yang, 2017; Yang et al., 2018; Yang et al., 2019). Finally, a lot of compounds were collected: (13E,17E,21E)-8-hydroxypolypodo-13,17,21-trien-3-one, (13E,17E,21E)-polypodo-13,17,21-triene-3,18-diol, (16S, 20R)-dihydroxydammar-24-en-3-one, (20R)-3β-acetoxy-16β-dihydroxydammar-24-ene, (20S)-3β,12β,16β,25-pentahydroxydammar-23-ene, (20S)-3β-acetoxy-12β,16β,25-tetrahydroxydammar-23-ene, (3R,20S)-3,20-dihydroxydammar- 24-ene, (8R)-3-oxo-8-hydroxy-polypoda -13E,17E,21-triene, 11α-hydroxypregna-4,17(20)-trans-diene-3,16-dione, 15α-hydroxymansumbinone, 16-hydroperoxymansumbin-13(17)-en-3β-ol, 1α-acetoxy-9,19-cyclolanost-24-en-3β-ol, 28-acetoxy-15α-hydroxymansumbinone, 2-methoxyfuranoguaia-9-ene-8-one, 35833-62-6, 3-methoxyfuranoguaia-9- en-8-one, 3β- hydroxydammar-24-ene, 3β-acetoxy-16β,20(R)-dihydroxydammar-24-ene, 4,17(20)-(cis)-pregnadiene-3,16-dione, 7β,15β- dihydroxypregn-4-ene-3,16-dione, beta-Sitosterol, Cabraleadiol monoacetate, Cabraleone, Chondrillasterol, Diayangambin, Epimansumbinol, Guggulsterol IV, Guggulsterol VI, Guggulsterone, Isofouquierone, Mansumbin-13(17)-en- 3,16-dione, Mansumbinoic acid, MOL001019, MOL001164, Myrrhanol C, Myrrhanone A, Myrrhanones B, Naringenin, Pelargonidin, Petunidin, Phellamurin, Quercetin, Stigmasterol, 3-oxo-tirucallic acid, Acetyl-alpha-boswellic acid, alpha-Boswellic acid, beta-Boswellic acid, Incensole, O-acetyl-α-boswellic acid, Phyllocladene, Tirucallol.

Since the application of biological models to predict XHP compounds has limitations (Metodiewa et al., 1997), in order to avoid missing active compounds during the pre-screening process, we searched a large number of references and selected oral absorbable compounds with pharmacological activity. Combined with relevant references (Huwez et al., 1998; Chen et al., 2013; Morikawa et al., 2017; Kunnumakkara et al., 2018; Hao, 2018), the following compounds are included: beta-Elemene, delta-Elemene, gamma-Elemene, Limonene, Curzerene, Germacrene B, Myrcenol, beta-selinene, Spathulenol, β-elemonic acid, 11-keto-β-boswellic acid, acetyl-11-keto-β-boswellic acid, trans-Anethole, ZINC01280365, SCHEMBL17727656, Oleanolic acid, Methyl deoxycholate, Deoxycholic Acid, Cholesterol, Cherianoine, Chenodeoxycholic acid, n-Nonane, Muscopyridine, Muscol, Estragole, Dihydroxanthyletin, Dihydroagarofuran, Cholesteryl ferulate, Androst-4-ene-3,17-dione, Allantoin.

#### Potential Targets of XHP

Four databases were utilized to predict the potential targets of XHP: Similarity ensemble approach (SEA) (http://sea.bkslab.org/) (Keiser et al., 2007), STITCH Database (http://stitch.embl.de/) (Kuhn et al., 2008; Kuhn et al., 2012), Swiss Target Prediction (http://www.swisstargetprediction.ch/) (Gfeller et al., 2014), and PubChem (https://pubchem.ncbi.nlm.nih.gov/) (Kim S. et al., 2019). The UniProtKB (http://www.uniprot.org/) was used for the correction of protein’s names and the collection of official symbols with the species limited to “*Homo sapiens*”. The details are described in **Table S1** (see Supplementary Material).

#### Triple Negative Breast Cancer Biological Network

To construct the biological network of TNBC, the TNBC-related genes were collected from OMIM database (http://omim.org/) and Genecards (http://www.genecards.org) (Zeng and Yang, 2017; Yang et al., 2018; Yang et al., 2019). Finally, one thousand and two hundred and twenty-one (1220) TNBC-related genes were obtained. These TNBC-related genes will be used for subsequent biological network construction and network analysis (see **Table S2**).

#### Human Transcriptomics Data

Transcriptome data come from GEO (https://www.ncbi.nlm.nih.gov/geo/). The data of *Olibanum* and β-boswellic acid intervention MD-MB-231 cells were obtained from GSE102891 (Mazzio et al., 2017).

### Network Construction and Analysis Methods

The data of protein-protein interaction (PPI) of XHP targets and TNBC genes were obtained from the String database (http://string-db.org/) with the species limited to “*Homo sapiens*” (Missiuro et al., 2009; Zeng and Yang, 2017; Yang et al., 2018; Yang et al., 2019). Then, these data were input into cytoscape software ver 3.7.0 (https://cytoscape.org/) for network construction (Bader and Hogue, 2003).

The networks were analyzed by the plugin MCODE to obtain cluster. The definition and the methodology of acquisition of clusters were described in our previous work (Zeng and Yang, 2017; Yang et al., 2018; Yang et al., 2019), such as “Exploring the pharmacological mechanism of Yanghe Decoction on HER2-positive breast cancer by a network pharmacology approach” (Zeng and Yang, 2017) and “Investigating the regulation mechanism of baicalin on triple negative breast cancer’s biological network by a systematic biological strategy” (Yang et al., 2019).

In addition, the DAVID database ver. 6.8 (https://david-d.ncifcrf.gov) were utilized to undergo Gene Ontology (GO) enrichment analysis and pathway enrichment analysis (Huang et al., 2009).

## Results and Discussion

### XHP’s Fingerprint

XHP and acetyl-11-keto-β-boswellic acid reference substance were analyzed and compared according to the steps of *HPLC Detection*. The fingerprint was shown in **Figure S1**.

### Experimental Results for MD-MB-231 Cell

#### Inhibition Effect of XHP on the Growth of MD-MB-231 Cell

**Figure 2:**
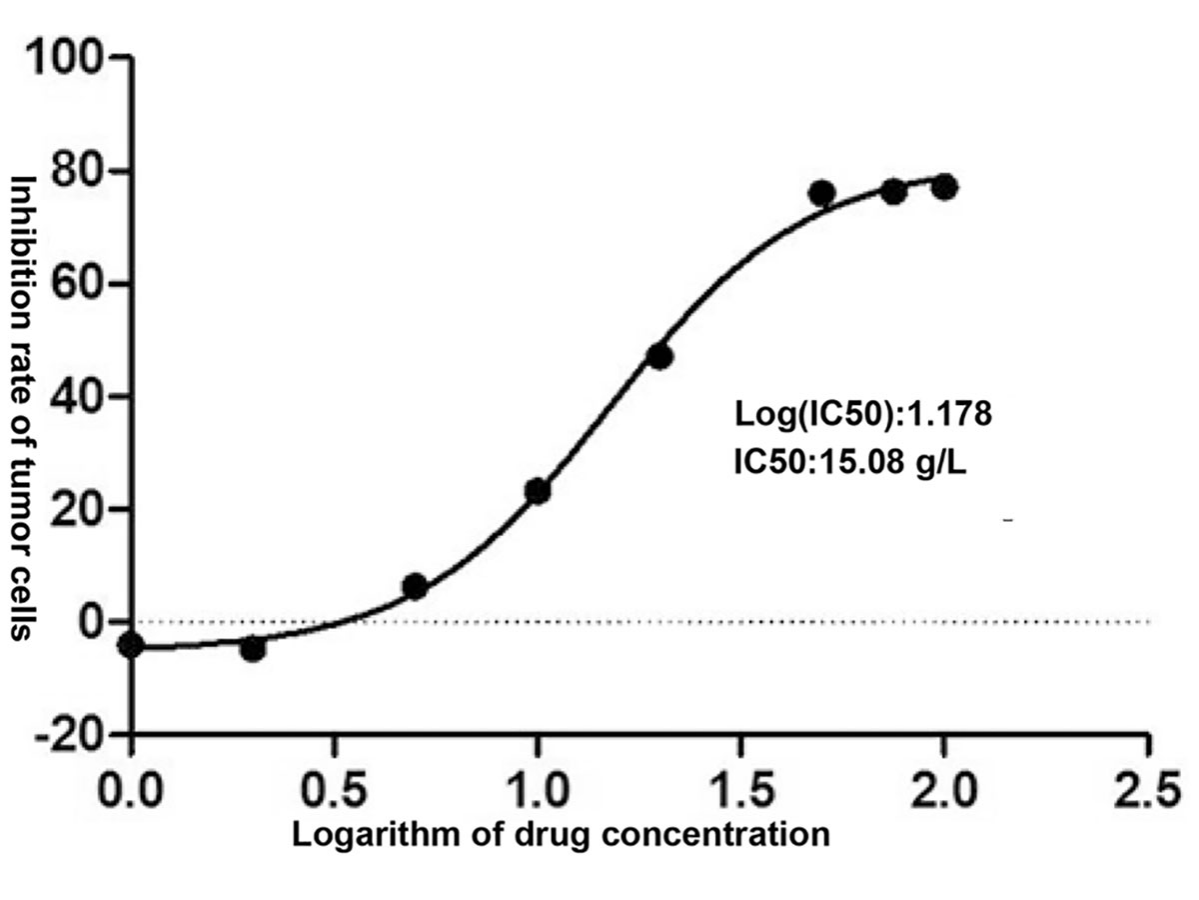
Inhibition rates of XHP on MDA-MB-231.

Different concentrations of XHP were applied to MD-MB-231 cells for 72 h, and the OD value of each group was detected by CCK-8 kit. The OD value could indirectly reflect the number of viable cells. The results are shown in **Table 2**. The XHP extract concentration value was logarithmically processed. The OD value of each concentration group was compared with the OD value of the non-medicated solvent group, and the cell inhibition rate was calculated. Draw a scatter plot with Log (concentration value) and cell inhibition rate (**Figure 2**). The IC50 value of XHP extract intervention in MD-MB-231 cells for 72 h was 15.08 g/L.

**Table 2:** Inhibition rates of XHP on MDA-MB-231.

XHP concentration (g/L)	OD 450nm	Inhibition Rates (%)
0	1.077 ± 0.008	–
1	1.119 ± 0.005	−3.954
2	1.127 ± 0.018	−4.660
5	1.008 ± 0.002	6.384
10	0.826 ± 0.002	23.288
20	0.569 ± 0.001	47.111
50	0.257 ± 0.004	76.096
75	0.254 ± 0.004	76.393
100	0.246 ± 0.001	77.149

#### Effect of XHP on the Viability of MD-MB-231 Cells

**Figure 3:**
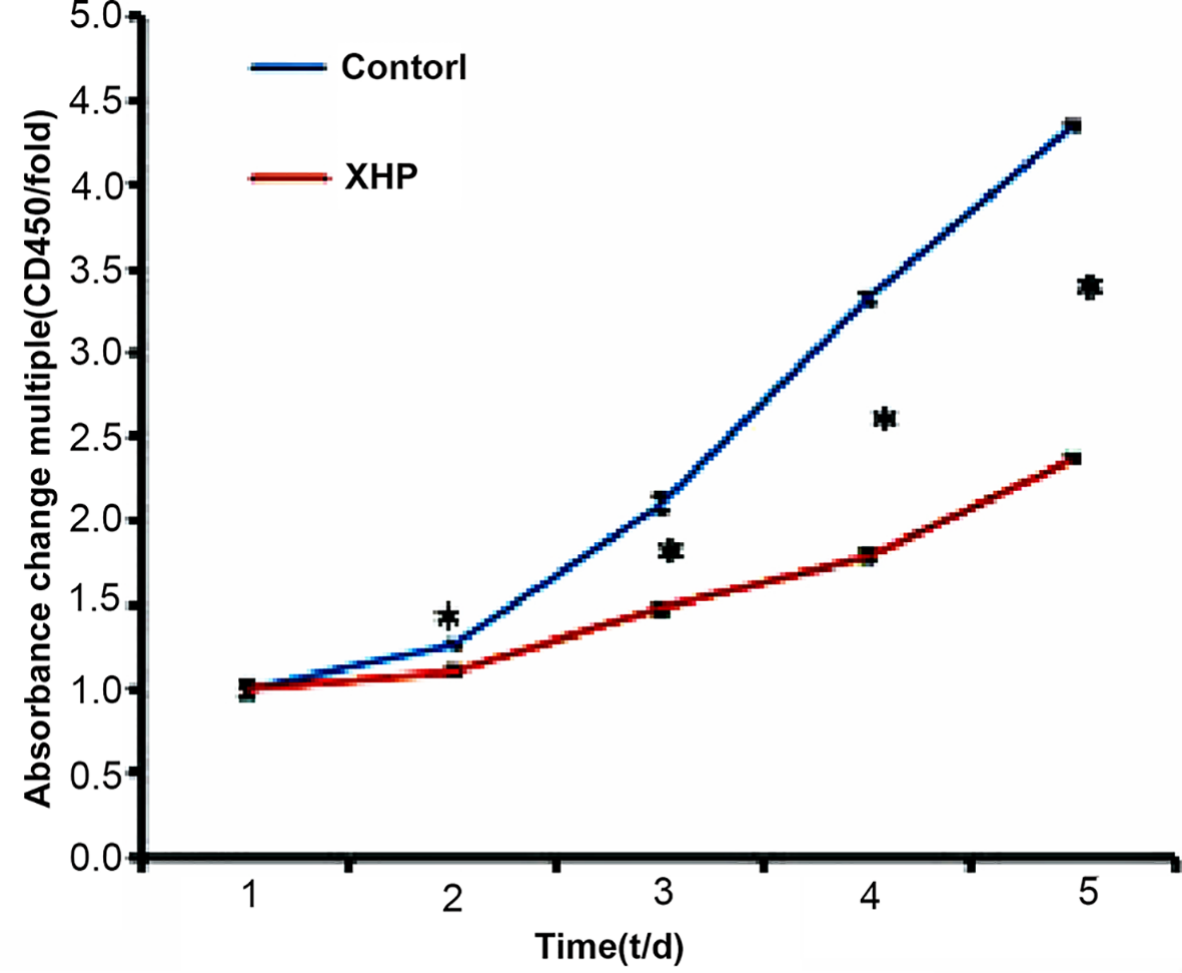
Inhibition rates of XHP on MDA-MB-231.

The CCK-8 reagent contains WST-8, which is reduced in the cell mitochondria to a highly water-soluble yellow formazan product, the number of which is proportional to the number of viable cells. The OD value measured at a wavelength of 450 nm can indirectly reflect the cell proliferation. The cells were intervened for 72 h with XHP extract at a final concentration of 15.08 g/L. Compared with the control group, the cell viability was significantly decreased (P < 0.01), as shown in **Figure 3**.

#### Effect of XHP on apoptosis of MD-MB-231 cells

**Figure 4:**
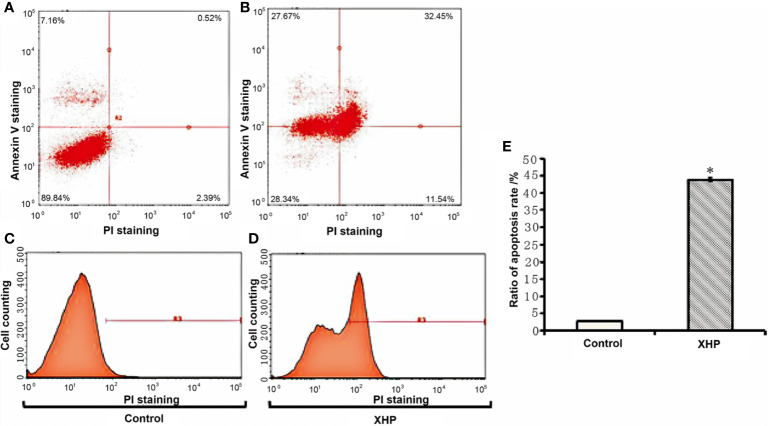
Effect of XHP on apoptosis of MD-MB-231 cells **(A, C)** are apoptotic maps of a random set of three duplicate wells in the control group; **(B, D)** are apoptotic maps of a random set of three duplicate wells in the XHP group; **(E)** is a histogram of the proportion of apoptotic cells in the two groups, compared with the control group; *P < 0.01).

The cells were intervened for 72 h with XHP extract at a final concentration of 15.08 g/L. Compared with the control group, the early apoptosis and late apoptosis of the cells after XHP intervention were significantly increased (P < 0.01) (**Table 3** and **Figure 4**).

**Table 3:** Effect of XHP on apoptosis of MD-MB-231 cells (x ± s).

Apoptosis	Control group	XHP group	P value
early apoptosis (%)	2.380 ± 0.056	11.623 ± 0.072	0.000
late apoptosis (%)	0.527 ± 0.012	32.243 ± 0.578	0.000

#### Effect of XHP on Migration Ability of MD-MB-231 Cells

**Figure 5:**
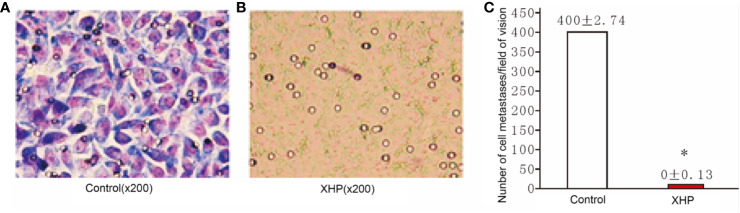
Effect of XHP on migration ability of MD-MB-231 cells **(A, B)** are the Giemsa staining maps of a random set of three duplicate wells in the control group and the XHP group, respectively; **(C)** is a histogram of the cell counts of the two groups. Each group of cells was randomly counted for nine fields of view; and take the average of the number of cells; compared with the control group, *P < 0.01.

The cells were intervened for 72 h with XHP extract at a final concentration of 15.08 g/L. The effect of XHP on cell metastatic ability was determined by observing the migration of cells in the Transwell chamber to serum-containing media. Compared with the control group, the migration ability of the cells after XHP intervention were significantly decreased (P < 0.01) (**Figure 5**).

#### Effect of XHP on Cell Cloning Ability

**Figure 6:**
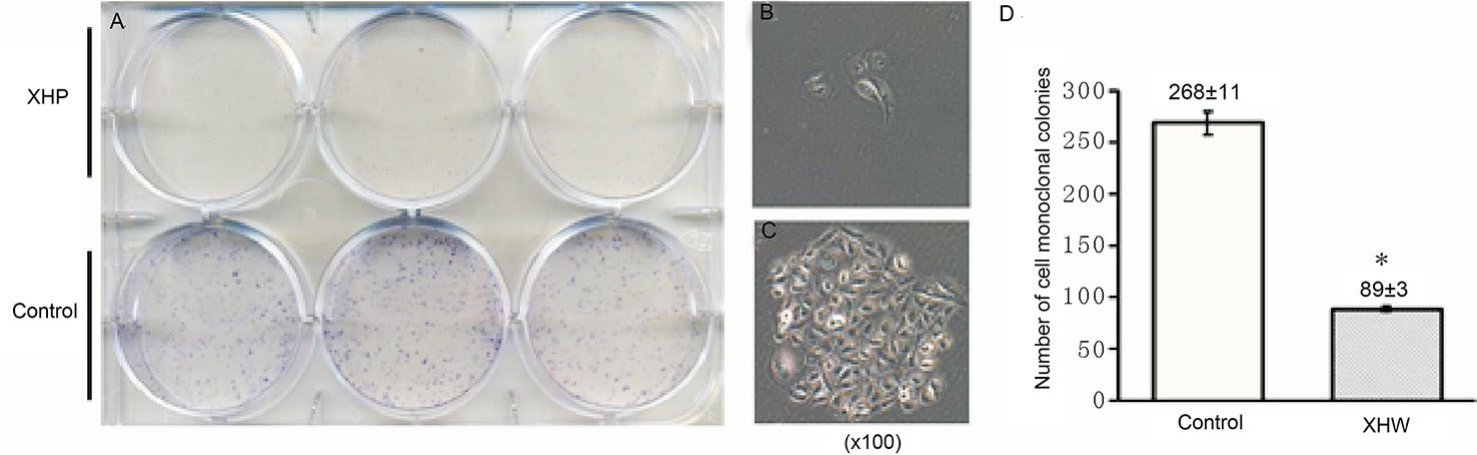
Effect of XHP on cell cloning ability. **(A)** is the 6-well plate culture plates after staining with crystal violet; **(B, C)** are the cell clones of a random set of three duplicate wells in the XHP group and the control group, respectively; **(D)** is the histogram of the number of cell clones in both groups; compared with the control group, *P < 0.01.

The cells were intervened for 72 h with XHP extract at a final concentration of 15.08 g/L. After 10 days of continuous culture, the clone formation rate was calculated to quantitatively analyze the proliferation ability of breast cancer cells. Compared with the control group, the cloning ability of the cells after XHP intervention were significantly decreased (P < 0.01) (**Figure 6**).

### Experimental Results for MDA-MB-453 Cell

#### Inhibition Effect of XHP on the Growth of MDA-MB-453 Cell

**Figure 7:**
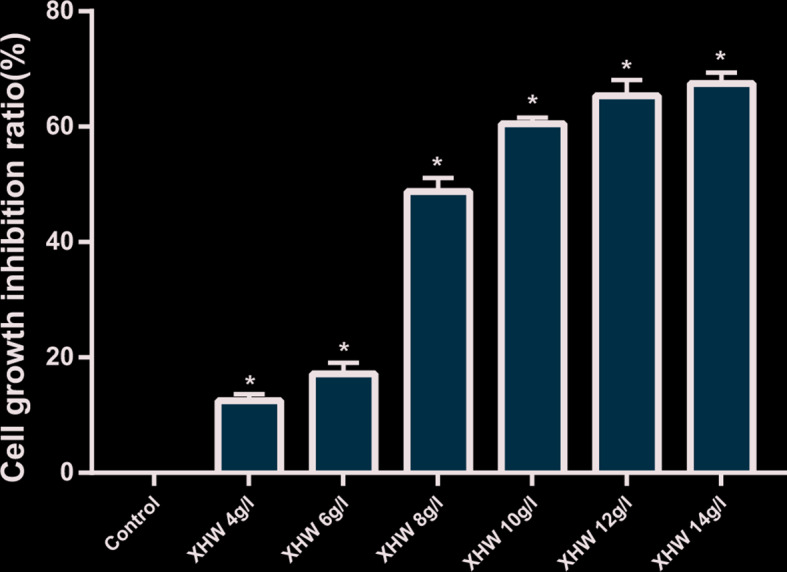
Inhibition rates of XHP on MDA-MB-453 Cell (* Compared with control group (0 g/L), P < 0.05).

The results of MTT also showed that XHP had a proliferation-inhibiting effect on MDA-MB-453 cells in a dose-dependent manner. When the concentration of XHP is lower than 6g/L, the inhibition rate of MDA-MB-453 cells is ≤20%, and there is no cytotoxicity. Therefore, 6 g/L was selected as the non-cytotoxic concentration of XHP for subsequent MDA-MB-453 experiments. The results are shown in **Figure 7**.

#### Effect of XHP on apoptosis of MDA-MB-453 Cell

**Figure 8:**
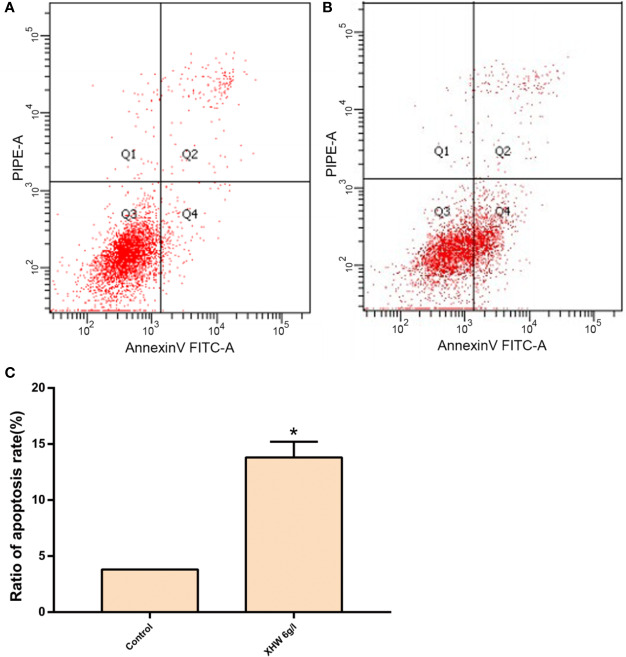
Effect of XHP on Apoptosis of MDA-MB-453 Cell. **(A)** apoptotic maps of a random set of three duplicate wells in the control group; **(B)** are apoptotic maps of a random set of three duplicate wells in the XHP group; **(C)** histogram of the proportion of apoptotic cells in the two groups, compared with the control group; *P < 0.05.

After 72 h intervention with 6 g/L XHP, the results of flow cytometry showed that the apoptosis rate in the test group was significantly higher than that in the control group (normal saline serum group) (P < 0.05) (**Figure 8**).

#### Effect of XHP on the Expression of Notch1, β-Catenin and c-myc mRNA

**Figure 9:**
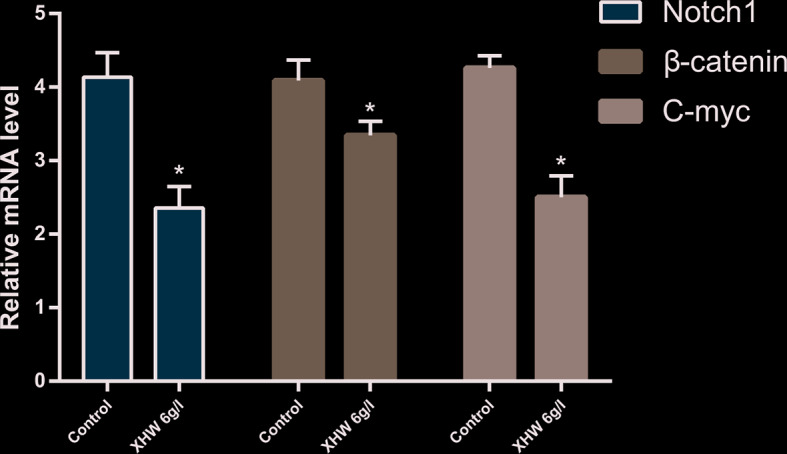
The Expression of Notch1/β-catenin/c-myc genes (*compared with control group, *<0.01).

Compared with the control group (saline saline group), the expressions of Notch1 mRNA, β-catenin mRNA and c-myc mRNA in the XHP group were significantly reduced (P < 0.01) (**Figure 9**).

This experiment showed that XHP can promote apoptosis, inhibit cell proliferation, metastasis and vitality, and can down-regulate the expression of Notch1 mRNA, β-catenin mRNA and c-myc mRNA at the cellular level. In addition, other studies have also demonstrated the anti-TNBC effect of XHP. Zheng et al. (2016) found that XHP significantly inhibited the viability of Hs578T cell line in a dose- and time-dependent manner. The mechanism may be that XHP induced apoptosis through the inherent Bcl-2 dependent pathway and cell cycle arrest. Su et al. (2018) found that XHP may promote Treg cell apoptosis in the tumor microenvironment and further inhibit the tumor growth of breast cancer in 4T1 mice. The mechanism may be that XHP up-regulated the MEKK1, SEK1, JNK1 and AP-1 gene and protein expression in Treg cells in the tumor microenvironment. Li et al. (2018a) further research indicates that XHP promotes apoptosis of Treg cells by inhibiting PI3K/AKT/AP-1 signaling pathway, thereby reducing the number of Treg cells, weakening the immunosuppressive state of the tumor microenvironment, reversing the mainstream immune escape, and inhibiting the growth of tumors. However, the molecular mechanisms of XHP’s anti-TNBC effects, such as regulatory pathways and targets, need further study. Based on this, this study will further study the mechanism of XHP reversing the multidrug resistance of tumors, and provide a theoretical basis for the development of new drugs and clinical combination drugs.

### XHP-TNBC PPI Network Analysis

#### XHP’s Potential Targets and TNBC Genes

After the potential target prediction, a total of 1,178 potential targets were obtained. *Myrrha* contains 550 potential targets; *Bovis Calculus* contains 680 potential targets; *Olibanum* contains 390 potential targets; *Moschus* contains 845 potential targets. Some of the targets contained in different herbs overlap (**Figure S2**). In the outer circle, red, blue, green, purple stand for potential targets of *Bovis Calculus*, *Moschus*, *Myrrha*, *Olibanum*, respectively. In the inner circle, the greater the number of purple links and the longer the dark orange arc, the more overlap between the input target lists. The blue link indicates the amount of functional overlap between the input target lists.

**Figure 10:**
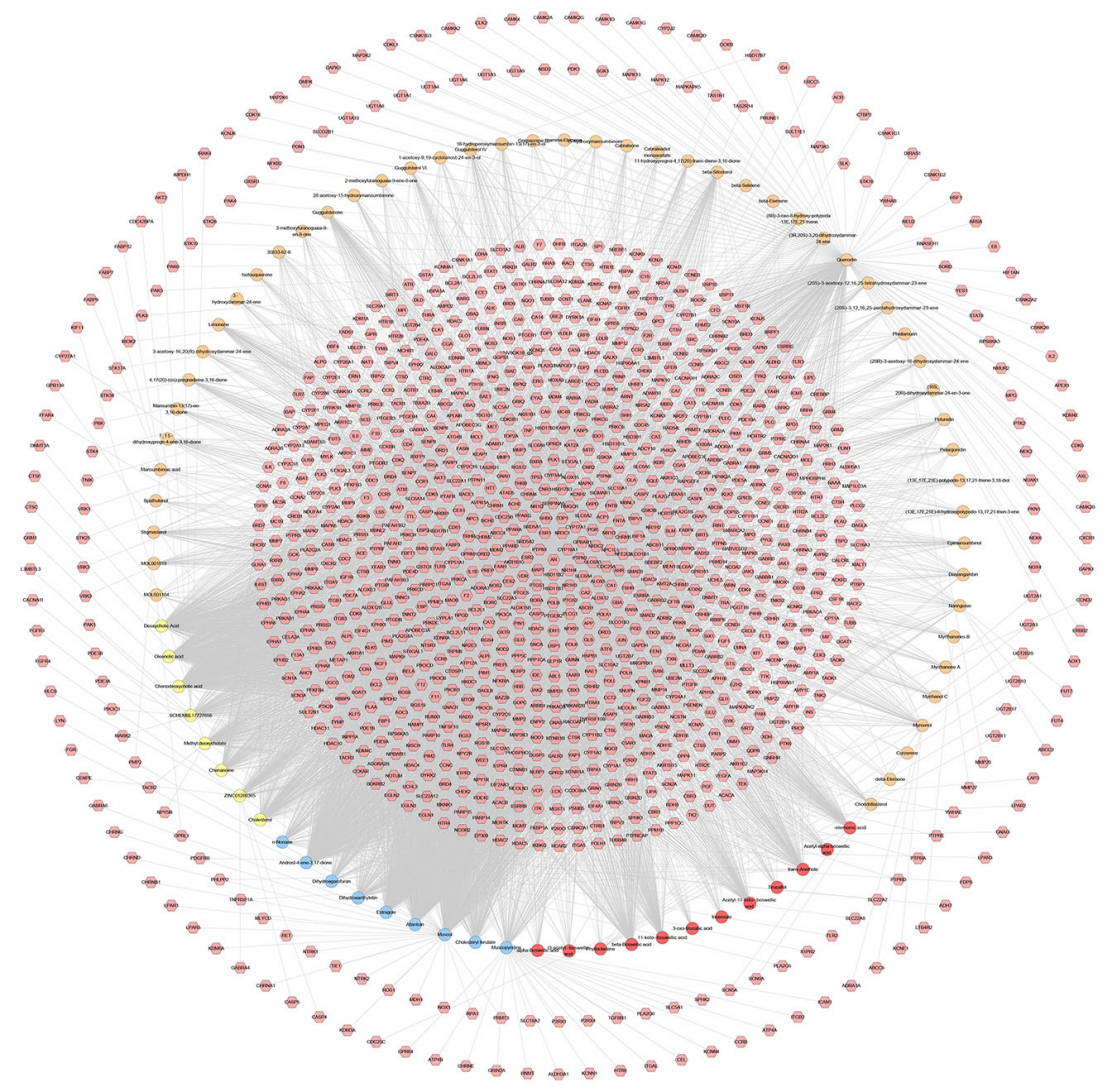
Potential compounds-potential targets network of XHP (The red circle stands for compound of *Olibanum*; the blue circle stands for compound of *Moschus*; the yellow circle stands for compound of *Bovis Calculus*; the orange circle stands for compound of *Myrrha*. The pink hexagon stands for potential targets.)

The relationship among potential compounds and potential targets was shown in **Figure 10**. This network consists of 81 potential compounds, 1,175 potential targets and 6,057 edges. The targets near the center are regulated by more compounds than ones in the peripheral. For example, the targets in the center are: AR (55 edges), CYP19A1 (52 edges), ESR1 (36 edges), NR3C1 (36 edges), ESR2 (35 edges), PTPN1 (35 edges), MAPT (34 edges), HSD11B2 (31 edges), NR1H4 (29 edges); the targets in the peripheral (AXL, CAMK2B, CXCR1, NEK2, NEK6) are regulated only by one compound. In addition, some important targets related to TNBC are regulated by more XHP compounds: EGFR (four edges), PARP1 (eight edges), PARP10 (three edges), PARP14 (one edge), PARP15 (one edge), PARP2 (two edges), VEGFA (two edges), PIK3CA (11 edges), AKT1 (eight edges), AKT2 (one edge). After searching, a total of 1,221 TNBC-related genes were obtained. These TNBC genes will be combined with XHP’s predicted targets to construct an XHP-TNBC PPI network so as to observe the association between XHP and TNBC.

It can be seen from the potential compounds-potential targets network of XHP that the herbs in XHP have multi-component and multi-target effects in anti-TNBC. Many compounds can act on one or more targets at the same time, and some targets can be regulated by multiple compounds. They may be the main active ingredient and target of XHP against TNBC. Currently, the study found that TNBC can also be subdivided into six subtypes: two basal-like-related (BL1 and BL2), two mesenchymal related subtypes [mesenchymal (M) and mesenchymal stem-like (MSL)], an immunoregulatory subtype (IM) and a tubular androgen receptor type (LAR) (Waks and Winer, 2019). LAR subtype is sensitive to androgen receptor (AR) inhibitors due to high expression of AR. Enzalutamide (an AR inhibitor) is an advanced prostate cancer drug approved by the US FDA (Traina et al., 2018). The current study on Enzalutamide is a phase II clinical study of patients with locally advanced or metastatic AR+TNBC; The study showed that approximately 55% of patients with TNBC were AR-positive, and enzalutamide had a good effect on the LAR subtype TNBC (Traina et al., 2018).

Recent studies have shown that tamoxifen may be effective against certain subtypes of TNBC, which is associated with ESR2 (Mukhopadhyay et al., 2019). Studies at the Roswell Park Comprehensive Cancer Center in the United States have shown that TP53 status is a determining factor in the duality of estrogen receptor-beta (ESR2) function (Mukhopadhyay et al., 2019). ESR2 and mutant TP53 can be combined to predict survival in patients with TNBC (Mukhopadhyay et al., 2019). Current research shows that miRNA genetic variation is associated with the expression of important receptors such as ER and HER2, and the single nucleotide polymorphism (SNP) site present in the estrogen receptor alpha gene (ESR1) may be involved in the development and progression of TNBC (Zhang, 2011).

Overall, AR, vascular endothelial growth factor (VEGF), poly(ADP-ribose) polymerase (PARP) and epidermal growth factor receptor (EGFR), PI3K, AkT, microRNAs and lncRNAs and so on are potential therapeutic targets for TNBC (Nagini, 2017; Chan et al., 2019 Nakhjavani et al., 2019).

#### XHP-TNBC PPI Network

**Figure 11:**
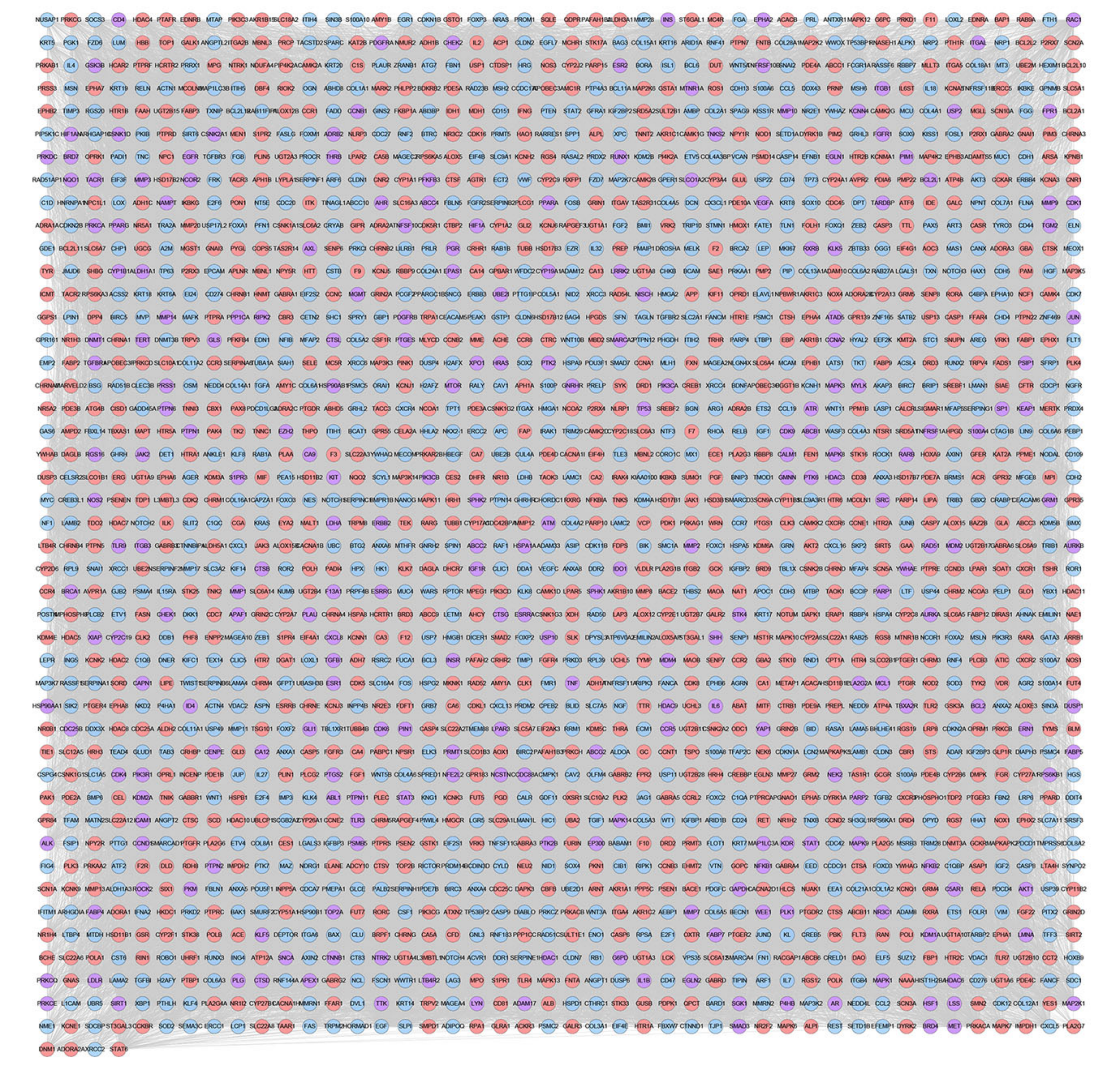
XHP-TNBC PPI Network (Purple circle stands for XHP-TNBC; Blue circle stands for TNBC genes; Pink circle stands for XHP targets.)

The relationship among XHP’s potential targets and TNBC genes were shown in XHP-TNBC PPI network. This network is composed of 247 XHP-TNBC targets, 907 XHP targets, 830 TNBC targets, and 73,021 edges. The top 20 targets of high-degree are selected and divided into three categories: (1) XHP targets: ALB (545 edges), CASP3 (420 edges); (2) TNBC genes: MYC (562 edges), EGF (468 edges), FN1 (460 edges), PTEN (446 edges); (3) XHP-TNBC: TP53 (743 edges), AKT1 (722 edges), GAPDH (662 edges), INS (608 edges), EGFR (594 edges), IL6 (530 edges), VEGFA (525 edges), MAPK3 (513 edges), SRC (460 edges), TNF (456 edges), HRAS (454 edges), MAPK1 (452 edges), STAT3 (441 edges), JUN (427 edges) (**Figure 11**).

#### Biological Processes of XHP-TNBC PPI Network

The XHP-TNBC PPI network were analyzed by MCODE to obtain the clusters. The clusters of this network were shown in **Figure S3** and **Table 4**. The genes and targets in clusters 1–10 were put into DAVID database to undergo GO enrichment analysis as an example.

**Table 4:** Cluster of XHP-TNBC PPI network.

Cluster	Score	Nodes	Edges	Targets and genes
1	84	84	3,486	CXCL16, HTR1E, GALR2, HRH3, CNR2, HRH4, FPR1, PTGDR2, BDKRB2, DRD4, MCHR1, LPAR5, CXCR3, LPAR1, GNAI1, CXCR6, CXCR4, CXCL8, HCAR2, NPY1R, NPY2R, CCR7, GABBR1, GPER1, CXCL1, S1PR3, CCR8, CNR1, S1PR4, GPR183, CCR1, CCR3, CCR2, GALR3, GNAI3, CHRM4, OPRK1, S1PR2, NPBWR1, LPAR2, DRD3, GPR55, MTNR1A, KNG1, CHRM2, PTGER3, CASR, GRM2, CXCR2, APLNR, GRM4, CCL5, ADRA2A, ANXA1, TAS2R31, APP, ADRA2C, ADRA2B, MTNR1B, HTR1A, CXCR1, DRD2, HTR1B, NMUR2, CX3CL1, TAS2R14, CCR5, TAS1R1, ADORA3, CCL19, C5AR1, ADORA1, CCR4, ACKR3, OPRM1, OPRD1, NPY5R, S1PR1, OPRL1, LPAR3, FPR2, CXCL5, CXCL13, HTR5A
2	80.154	144	5,731	HIF1A, AKT1, STAT3, PTEN, KRAS, CDH1, MKI67, PROM1, TLR4, CTNNB1, NR3C1, CASP3, MYC, PARP1, TGFB1, WNT5A, IGF1R, GAPDH, MET, ZEB1, SNAI1, CASP1, ITGB1, IL6, NOTCH1, MTOR, RUNX2, NES, RB1, SNAI2, TIMP1, JUN, PTPRC, FOXM1, STAT1, MMP9, CASP8, INS, HSPA4, CDKN2A, TLR2, MAPK9, FOXP3, CCNA2, CCND1, BCL2L1, ALB, TNF, RAF1, MAP2K1, MMP14, PPARG, CDKN1A, PLAU, BCL2L11, HGF, MMP13, NGF, MMP3, ICAM1, CAV1, CDK4, SHH, TLR7, SRC, XIAP, MAPK1, NLRP3, CDKN1B, CD44, SPP1, CREB1, EPCAM, IL2, JAK2, ABL1, PTGS2, PTPN11, CCNB1, CCL2, TLR3, BECN1, TERT, WNT1, SERPINE1, POU5F1, MAPK3, CDH2, TNFRSF1A, SMAD3, MAPK8, MAP2K7, PLG, LEP, WNT3A, MMP1, BDNF, SOX2, HRAS, EGR1, FOS, FGF2, CDC42, NOS3, MAPK11, RHOA, HDAC1, EP300, AKT2, JAG1, RPS6KB1, CASP9, IL4, SOCS3, MAPK14, HSP90AA1, ERBB3, TWIST1, GSK3B, CSF1, ANXA5, HMOX1, NANOG, JAK1, IFNG, SMAD2, MPO, CCND2, ABCG2, TLR9, LOX, MMP7, CSF1R, IL1B, TNFSF11, BRCA1, EGFR, AR, ATM, ESR1, RELA, MAPK10, TP53, PGR
3	42.4	51	1,060	FFAR1, PIK3CA, GCGR, CCKBR, TACR3, CCKAR, PTGER1, HTR2A, PIK3R3, PLCB2, TACR1, PIK3R1, PTAFR, PTGFR, NPSR1, GNRHR, FFAR4, GRM5, ADRA1A, EDNRA, EDNRB, KISS1, LTB4R2, CHRM5, CHRM1, MCL1, CHRM3, F2, MDM2, AGTR1, HTR2B, FOXO3, PLCB3, GNRH2, KDR, GRM1, EDN1, KISS1R, AVPR1A, HCRTR1, HRH1, TBXA2R, HTR2C, F2R, OXTR, NTSR1, HCRTR2, TRHR, MMP2, LTB4R, TACR2
4	28.58	239	3,401	CD274, ESR2, MSLN, VEGFA, SELE, ADAM17, MUC1, KRT5, NFKB1, FAS, FGFR2, GHRHR, LGALS3, EZH2, GLI1, HMGA2, MFGE8, FN1, IDO1, A2M, ALDH1A1, AKT3, ERCC1, SIRT1, SHC1, KIT, NEK2, PTK2, AREG, HSPA5, FGF1, CCNE1, DNMT1, TYMS, CDK2, CCNE2, PDGFRB, JAK3, FGFR1, GPBAR1, IGF2, H2AFX, CLU, KRT18, ELANE, ILK, HBEGF, PDGFRA, CCNB2, CDK6, CD24, KRT14, ALK, NRAS, PTHLH, PTPN1, NOS2, TTK, PLAUR, PTGER2, CDH5, VIM, PTGIR, EPHA2, VWF, NT5E, PTGER4, ETS1, GIPR, PTGDR, SERPINC1, IGF1, AURKB, DICER1, LAMB1, COL2A1, COL4A1, FGB, HTR4, SERPING1, SKP2, COL3A1, SOX9, VCAN, LGALS1, SERPINH1, COL4A3, EGF, FBN1, F13A1, ADIPOQ, COL11A2, COL5A1, TEK, AVPR2, SYK, KIF14, F3, HTR7, CDK1, RAD51C, EIF4E, LTBP1, CRHR1, MC4R, SERPINF2, MC1R, MC5R, LAMB2, ACE, BMI1, TNC, COL18A1, COL4A6, COL6A2, COL6A3, IGFBP3, OSM, LAMC1, VEGFC, COL11A1, ERBB4, COL6A1, COL7A1, CCND3, HPGDS, MELK, PTTG1, COL4A2, HRG, COL14A1, ERCC5, CDC20, COL4A5, XRCC1, KIF11, COL12A1, COL13A1, COL5A2, COL8A2, ADORA2B, CALCRL, ADRB2, NFKBIA, NOTUM, DUSP1, IL7, NFKB2, SLC2A1, FGFR3, ITIH2, ITGA6, ACTN1, COL15A1, COL8A1, ALDOA, MMRN1, COL16A1, GPR84, GNAO1, ACTN4, TXN, COL21A1, COL24A1, HTR6, HSP90B1, LCK, COL5A3, COL6A5, COL28A1, COL6A6, MEN1, KIFC1, MSH6, PGF, DKK1, FLT1, LYN, RXFP1, ARG1, NOX4, PRKCZ, TGFB2, FASLG, GADD45A, NFE2L2, ZEB2, STAT6, MMP10, KRT8, IGFBP1, APAF1, ARRB1, DRD1, KLF4, TSHR, XBP1, RAD51AP1, CRHR2, E2F1, MAP2K2, HMGB1, RELB, IL18, PLK4, ITGAX, RAD54L, PBK, TGFBR1, ABCB1, GLP1R, SP1, SOD2, RAD50, GNAS, CGA, RACGAP1, TAAR1, PDIA6, WRN, AXIN1, KRT19, GHRH, ADAM10, ANGPT1, HSPB1, GPR32, PTH1R, TJP1, SMAD7, GINS2, CFD, NUSAP1, SDC1, NF1, CENPE, GMNN, ERBB2, P4HA1
5	17.098	174	1,479	BRCA2, CTSC, CDC7, HSPA1A, CD276, XRCC2, S100A7, CHEK2, PALB2, BCL2L2, RET, BCL2A1, NTRK1, SOX10, TUBB, PRKDC, AHR, PSMC2, CDK5, GATA3, PSMC4, PMAIP1, PKM, VDR, ANXA2, EHMT2, KDM6A, SREBF1, AXL, RAD51B, CASP7, NOX1, HIST1H2BA, CDC45, NCF1, TP53BP1, SIN3B, TNFRSF10B, RAD51, EIF2AK3, BORA, MMP11, RIPK1, GLA, VCP, CTSA, TNFSF10, ANGPT2, CCRL2, NOD2, FANCM, APEX1, TGFBR2, BIRC5, BRIP1, AURKA, BIRC2, SUZ12, NTRK2, RAC1, DBF4, CYP19A1, NOTCH2, FANCA, PARP2, NQO1, CDKN2B, STAT2, GUSB, XRCC6, NTF3, CCNA1, CDC25A, MAP1LC3B, MSH2, CDCA7, FOXA2, WT1, FABP5, SIRT6, RICTOR, FUCA1, ARSA, FADD, OGG1, BIRC3, CTSB, XRCC3, MAP3K5, MAPK12, MAPK13, NGFR, CDK7, ECT2, HDAC2, HDAC8, MCAM, HDAC11, CA9, HDAC4, CCT2, HDAC10, TTR, TOP2A, PTK2B, PSMD14, DIABLO, POLH, FOSL1, DCN, PSMB5, IDH1, RPTOR, SDCBP, PLK1, PRKCD, LCN2, CTSG, TACC3, TNFRSF11B, TUBB4B, SIN3A, IMPDH1, GLI2, FCGR1A, IRAK1, FGF22, RRM1, AXIN2, BAX, RAD52, BSG, CDC25C, GRN, RBBP8, LMNA, ELN, FBXW7, ATR, FRK, MAPK7, E2F4, IKBKB, POSTN, FLT3, INCENP, E2F6, SMARCA4, CASP6, RASSF1, UHRF1, CHEK1, KAT2B, PSMA4, VTN, ATF6, MMP8, RAD23B, CREBBP, KEAP1, PI4K2A, ERCC2, XPC, ROCK1, PRL, ATG7, AGER, XRCC4, WEE1, PSMC5, LGR5, NOTCH3, PSMC1, SETD1A
6	9.333	121	560	HSPA8, SETD1B, CALR, FGFR4, BCL2, SMARCA2, RNF41, KDM1A, RPA1, SFN, NRP1, VDAC1, NID2, TBL1X, PIK3CB, YAP1, TIMP3, FBXL14, REST, HSP90AB1, NEDD4L, CTNND1, FUT4, MMP12, MAP3K7, CD38, INSR, RPS6KA1, BLM, DNMT3B, WNT10B, LDLR, MECOM, TP73, CYLD, TGFA, PRKCA, MDM4, BMP6, MITF, STMN1, BACE1, YES1, HDAC9, CALM1, KAT2A, KLF5, CDC25B, HK1, EPHB2, SIAH1, SERPINA1, ITGAL, COL1A1, ELAVL1, CDK9, TOP1, RNF4, FGA, FGG, ITGB3, MGMT, LEPR, NCOA1, BID, DET1, POLK, TGM2, GAS6, PLCG1, FEN1, HSPG2, HDAC7, IKBKG, PRKCB, HDAC6, SUMO1, PIK3CD, PIK3CG, WNT5B, IRAK4, PAK1, PLK2, HDAC3, SGK1, SIRT2, UBE2M, PDCD1LG2, UBE2I, DUT, THBS2, H2AFY, MLH1, CDC27, BCL6, DROSHA, MX1, RBBP4, ATF2, MBD2, UBE2B, PDCD1, ARID1A, TBL1XR1, EZR, PEBP1, S100A4, FOXA1, EED, JUNB, RNF2, SMURF2, H2AFZ, JUND, BCL3, PSEN1, YWHAG, RBBP7, NID1, NR2E1, BARD1
7	7.827	163	634	CTSH, RASA1, THPO, CDK12, P4HB, CCNC, MPHOSPH8, ITGB4, GLI3, YWHAE, PRLR, FKBP1A, HSPD1, HBB, POLA1, ATIC, PCGF2, YWHAZ, TIPIN, PLA2G6, CANX, CHD4, HTT, TP63, ITGB2, MAP3K3, RUNX3, PDCD4, NOD1, MAPT, FZD7, DHFR, SPHK1, PLA2G5, UBC, PTPN2, SLC6A4, FOXC1, PTGS1, KDM4A, FTH1, YBX1, ATAD5, OLFM4, QPCT, CCNB3, CAPN1, NME1, PTPN6, TDP1, NAMPT, SNCA, RUNX1, EFNB1, LRP6, FURIN, MAP1LC3A, LTF, PRMT1, RPS6KA3, DVL1, CES1, TRIM28, NUMB, ETS2, EPHA5, YWHAB, NOTCH4, DDB1, NR1I2, SULT1E1, RORC, KRT20, UGT1A3, UGT1A9, UGT1A4, PRKACB, USP1, FABP4, GSTP1, CYP2J2, PPARA, ISL1, NCOA3, PLA2G4A, XPO1, APC, POLI, RIPK2, COL1A2, SMC1A, HDAC5, FSCN1, CDK16, CSTB, ITGA5, ARNT, PRKCE, NOS1, PLK3, WNT11, TOP2B, CYP2C9, PIK3C3, DUSP6, FOSB, FZD6, CYP2B6, TGFBI, ETV4, PDPK1, CTSL, FBLN1, PLA2G2A, TAB3, SIRT5, F9, MALT1, LAMA4, MSN, CTSD, BAK1, SPARC, BRD4, RAPGEF3, PLA2G3, SLCO1B1, MME, RARA, LOXL1, SFRP1, NCOR1, LTA4H, IGFBP2, TYK2, HGS, BGN, ALOX15, ALOX12, ALOX15B, CSNK2A1, ALOX12B, ROCK2, BRMS1, CEACAM5, PRSS3, SH3GL1, NCOR2, LUM, CTSK, IFNA2, ITIH4, FANCF, G6PC, BNIP3, AKR1B1, CTSS, AMBP, TRIP10, PRMT5, PRKACA, FASN, DNMT3A
8	5.475	62	167	HSD17B2, PRKAR2B, UGT1A6, UGT1A8, UGT1A1, KPNB1, MAP3K2, CDK8, AKR1C3, RARB, HSF1, AKR1C1, F11, CHRNA1, CYP3A4, MAP2K6, GC, STK10, HSD3B1, YWHAQ, HSD17B12, P2RX1, ABCC2, LAMC2, TLN1, SRD5A1, PHLPP2, CYP2C18, ACACA, ITGA2B, THRA, MAPKAPK2, F7, HSD17B1, NEDD4, CSNK1A1, RXRA, F10, SLCO1B3, CHRNA4, CES2, ADAM8, GSK3A, LRRK2, JUP, CDK11B, TUBA1A, UBE2D1, HMGCR, CHRNB4, CCNT1, RAB27A, LAMA5, CD81, DPP4, CETN2, HSD17B3, SCD, NR1I3, ARF6, TRPM2, THRB
9	5.194	73	187	PPP1CA, SLCO1A2, PON1, CD74, CAMK2D, MAOB, FGR, KDM2A, ROS1, GABRA6, RXRB, ABCB11, SLC18A2, HNMT, EPAS1, GABRA2, ALDH3A1, CD47, CD4, PHF8, KDM5C, KDM5B, GABRA4, RARG, NCOA2, PTPN22, CLIC1, COPS5, SLC6A2, S100A9, PELP1, CLIC5, CYP1A2, SLC6A3, ERN1, SERPINB6, NR2F2, CYP1A1, NLRP1, GAA, RXRG, GABRB3, GSTA1, SLIT2, AOX1, CYP1B1, ITGAV, PRKCQ, LDHB, MST1R, S100A8, EPHB6, FLNA, P2RX7, GABRA3, PITX2, GABRA5, NKX2-1, MAOA, GLRA1, HSD17B7, TXNIP, PTK6, EPHA10, EPHA7, SEMA3C, EPHA4, EPHA8, EPHB3, EPHB1, CUL4A, EPHA1, EPHA6
10	5	5	10	CREB5, CREB3L1, PRKD3, PRKD2, PRKCG
11	4.857	22	51	EPHX1, CYP2D6, GLUL, DPYD, ADH1A, ADH1B, KL, ADH1C, PLIN1, CYP2A6, FABP3, CPT1A, CYP26A1, TYMP, PGD, LPIN1, ACACB, LIPE, UGT2B17, UGT2B15, MDH1, TKT
12	4.25	9	17	KCNH2, KCNJ1, KCNK9, ABCC9, KCNE1, KCNN1, KCNMA1, KCNK2, TNNT2
13	4	34	66	EIF4H, CACNA1B, ACHE, NR3C2, PTBP1, MT3, CHRNG, MGLL, CHRNB1, CHRND, HSD11B1, ENO1, SLC22A1, CYP11B1, CHRNA3, EIF4A1, CHRNE, CYP11B2, GRIN1, SCN2A, CYP2C19, G6PD, EGLN2, FAAH, CYP2E1, CYP2C8, ALDH1A3, GCK, CHRNA7, CYP2A13, CASP5, HKDC1, PYGL, ADH7
14	4	8	14	PSENEN, APH1A, APH1B, PAFAH1B2, PAFAH2, PAFAH1B3, NCSTN, PSEN2
15	4	5	8	ANXA6, AHNAK, S100A6, ANXA4, ANXA3
16	3.93	58	112	CLDN2, C1S, CYP51A1, UGT1A10, SMARCD3, CAMK2G, HIF1AN, ESRRA, AKR1C2, EIF4G1, EGLN3, SREBF2, PAK4, C1QA, GRIN2C, GRIN2A, GRIN2D, UGT2B7, CBFB, PINK1, SLC12A5, DNM1, AGRN, C1QB, MTHFR, SRD5A2, C1QC, NR0B1, FDPS, SQLE, NR1H2, RPL39, GABRB2, MAPK6, LDHA, CLDN7, GSTO1, RPL9, CBR3, SLC16A3, CLDN3, MYLK, EEA1, TFAM, MPEG1, CYP17A1, LSS, GABRA1, PFKFB3, SHBG, TSG101, DHCR7, SORD, MUC4, FABP1, TFF3, FDFT1, ACSS2
17	3.846	14	25	PPP5C, HNRNPA1, AHCY, BTRC, LMAN1, RPSA, RAN, LMAN1L, FOLR1, RAB1B, NAE1, UBE2N, NCL, RAB1A
18	3.333	4	5	UCHL3, UCHL5, UBLCP1, USP13
19	3	3	3	CDH13, DAPK1, HIC1
20	3	3	3	BRD7, BRPF1, ING5
21	3	3	3	LOXL2, MFAP5, MFAP2
22	3	3	3	CSNK1G2, CSNK1G3, CSNK1G1
23	3	9	12	DAO, SLC6A7, SCN9A, P2RX3, SCN10A, EPHX2, TRPV4, GSTK1, GABRD
24	3	3	3	UBA2, CTBP2, TLE3
25	3	3	3	NAAA, DAGLA, DAGLB
26	3	3	3	KCNA3, KCNN4, SCN5A
27	3	3	3	RAB9A, VPS35, RAB25
28	3	3	3	KCNA5, PLEC, IMP3
29	2.923	14	19	GRIN2B, NODAL, PRDM14, CAMK2A, PPP1CC, PAX8, PIP, FOXD3, PTPN5, TFAP2C, SIX1, CFTR, SCN1A, GABRG2
30	2.762	22	29	GLS, CAMK2B, DGAT1, CA2, ABCC1, SLCO2B1, SLC16A4, PFKFB4, FABP2, MPI, PDE3B, GFPT1, SLC29A1, ATP12A, ATP4A, PRKAA2, ADCY10, SLC10A2, PDE4B, PDK1, MLYCD, SLC22A8
31	2.667	4	4	NEU2, FUT7, ST3GAL3, ST3GAL1
32	2.667	7	8	AMY1C, AMY1B, KLK5, CASP14, CA6, CYP24A1, KLK7
33	2.5	5	5	HSPA9, SIGMAR1, BAG4, PABPC1, EIF2S1

After the GO enrichment analysis, a lot of biological processes of each cluster were return. Cluster 1 is associated with chemokines and their receptor-mediated signaling pathways, immune responses, ERK1/2 signaling pathways, angiogenesis, T cell chemotaxis. Cluster 2 is involved in apoptosis and cell proliferation, hypoxia induction, cell cycle, estrogen-mediated biological processes, canonical Wnt signaling pathway, ERK1/2 signaling pathway, angiogenesis, T cell-mediated immune response. Cluster 3 is involved in ERK1/2 signaling pathway, MAPK signaling pathway, PI3K signaling pathway, angiogenesis, and inflammatory response. Cluster 4 is involved in cell migration and adhesion to angiogenesis, extracellular matrix, hypoxia-induced, PI3K signaling pathway, MAPK signaling pathway, ERK1/2 signaling pathway, and endoplasmic reticulum stress. Cluster 5 is associated with tumor necrosis factor-mediated signaling, apoptosis, cell cycle, angiogenesis, T cell receptor signaling pathway, Wnt signaling pathway, and endoplasmic reticulum-mediated endogenous apoptotic signaling pathway. Cluster 6 is involved in transcriptional regulation, apoptosis, cell matrix adhesion, T cell receptor signaling pathway, angiogenesis, Wnt signaling pathway, cell cycle, immune cell chemotaxis and T cell costimulation. Cluster 7 is associated with apoptosis, extracellular matrix, negative regulation of Wnt signaling pathway, NF-κB signaling pathway, and angiogenesis. Cluster 8 is related to redox, steroid metabolism, androgen and estrogen-mediated biological processes, oxidative stress. Cluster 9 is associated with redox, chemokine generation and mediated biological processes, steroid hormone-mediated signaling, angiogenesis, T cell selection, cell migration. Cluster 10 is involved in the transmission of intracellular signals. The details of each cluster and biological process was described in **Table S3**.

**Figure 12:**
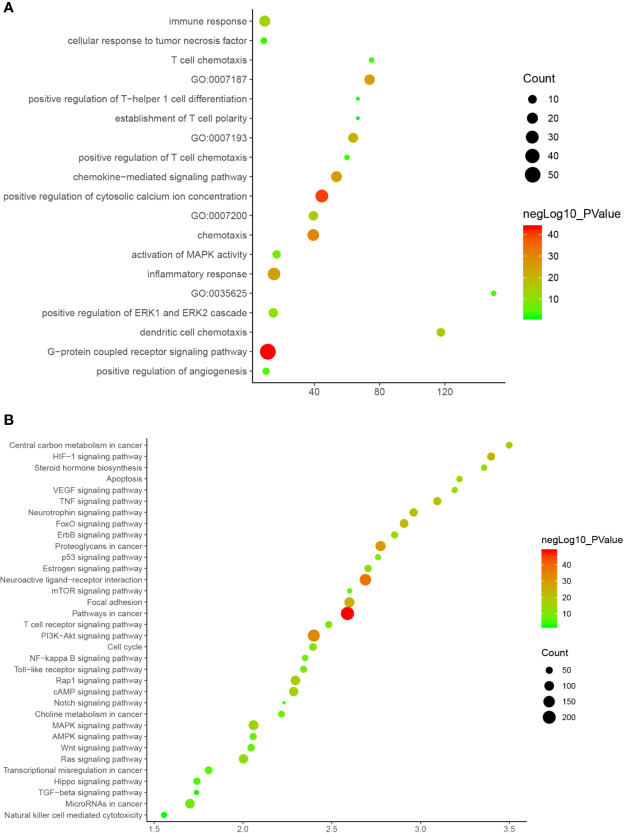
Bubble chart **(A)** biological processes; **(B)** signaling pathway. X-axis stand for fold enrichment.

Since Cluster 1 contains many classic biological processes, bubble chart is created using the main biological process data contained in Cluster 1 (**Figure 12A**).

#### Signaling Pathways of XHP-TNBC PPI Network

**Figure 13:**
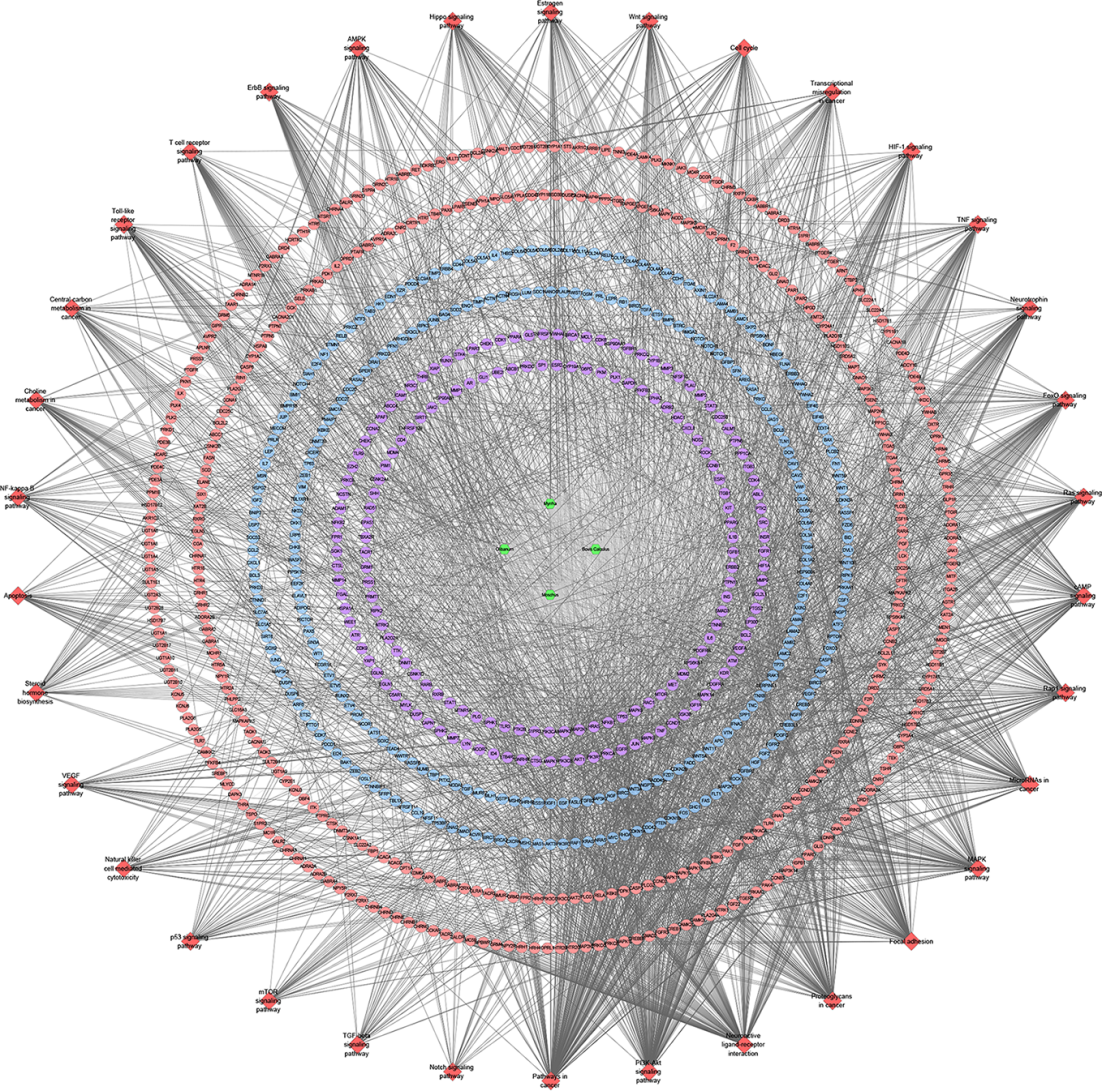
Signaling pathway of XHP-TNBC PPI network (Red diamond stands for signaling pathway; Purple circle stands for XHP-TNBC; Blue circle stands for TNBC genes; Pink circle stands for XHP targets).

The XHP targets combining with TNBC genes were put into DAVID database for pathway enrichment analysis. After this, thirty-four (34) anti-TNBC-related signaling pathways were returned (**Figure 13**).

These signaling pathways are ranked according to the degree of enrichment (negative correlation with P value) and count from large to small. The top 10 is: Pathways in cancer (P= 5.47 * 10^−49^; Count = 209), Neuroactive ligand–receptor interaction (P = 4.54 * 10^−38^; Count = 153), PI3K-Akt signaling pathway (P = 5.01 * 10^−34^; Count = 170), Proteoglycans in cancer (P = 6.38 * 10^−30^; Count = 114), Focal adhesion (P = 1.22 * 10^−25^; Count = 110), HIF-1 signaling pathway (P = 1.10 * 10^−24^; Count = 67), FoxO signaling pathway (P = 8.15 * 10^−23^; Count = 80), TNF signaling pathway (P = 1.43 * 10^−21^; Count = 68), Neurotrophin signaling pathway (P = 1.60 * 10^−21^; Count = 73), Rap1 signaling pathway (P = 3.91 * 10^−18^; Count = 99) (**Figure 12B**). The details of each signaling pathway was described in **Table S4**.

Through the above network analysis, we found that *Myrrha*, *Bovis Calculus*, *Olibanum* and *Moschus* in XHP has anti-TNBC effect. Among them, the main compounds of anti-breast cancer in *Olibanum* are boswellic acids, and their targets are basically the same. Among them, CYP19A1, ESR1, NR3C1, ESR2, PTPN1, MAPT, PTPN2, SHBG and so on are the main common targets. The main anti-breast cancer chemical compounds of *Olibanum* are pentacyclic triterpenoids, such as beta-Boswellic acid, Acetyl-11-keto-β-boswellic acid, 11-keto-β-boswellic acid, Acetyl-α-boswellic acid, O-acetyl-α-boswellic acid and so on (see **Figure S4**).

Meanwhile, the main component of *Myrrha* are Quercetin, beta-Sitosterol, Guggulsterone, Limonene, Myrcenol. the main component of *Bovis Calculus* are Chenodeoxycholic acid, Deoxycholic Acid, Oleanolic acid, Cholesterol, Methyl deoxycholate. The main component of *Moschus* are Androst-4-ene-3,17-dione, Allantoin, Muscol, Estragole, Cholesteryl ferulate. The targets mainly regulated by them also include CYP19A1, ESR1, NR3C1, ESR2, PTPN1, MAPT, PTPN2, SHBG and so on.

At present, the research direction of TNBC therapeutic drugs is mainly three aspects: new targeted therapy, immunotherapy and new endocrine therapy (Lehmann et al., 2016; Garrido-Castro et al., 2019). The first aspect is the new targeted therapy, which is developed to reduce toxicity, reduce the risk of disease progression, and improve patient prognosis. A variety of targeted drugs have entered the clinical trial phase. The targeted treatment of TNBC is divided into four major categories (Lee et al., 2019; Nakhjavani et al., 2019). (1) Targeted therapy for DNA repair: The main research direction is poly ADP-ribose polymerase (PARP), a key enzyme involved in DNA repair, which recognizes breakpoints in DNA single strands and repairs (Lord and Ashworth, 2017; Caulfield et al., 2019). The BRCA gene (BRCA gene mutation detected in 11.2% of patients with TNBC) is sensitive to PARP inhibitors (Evans et al., 2017; Densham and Morris, 2019). Among them, olrapani combined with chemotherapy drugs achieved a high objective response rate in clinical trials. However, due to its mechanism of inhibiting DNA repair, patients are more likely to develop a second primary tumor (Parker, 2011). (2) Targeted therapy for tyrosine kinase inhibition (TKIs): TKIs are a class of drugs that inhibit the activity of tyrosine kinases (Smith et al., 2004), which blocks the downstream signaling pathway by inhibiting the phosphorylation of protein tyrosine residues, thereby inhibiting tumor growth and metastasis (Arora and Scholar, 2005). Both epidermal growth factor receptor (EGFR) and vascular endothelial growth factor receptor (VEGFR) have receptors for tyrosine kinase activity, which can control the proliferation, invasion and metastasis of tumor cells by regulating various signaling pathways (Ayeni et al., 2015; Jayson et al., 2016). However, existing studies have shown that EGFR inhibitors alone are difficult to achieve anti-tumor effects (Ueda et al., 2017). Our transcriptomics and chemical informatics studies have shown that *Olibanum* can target EGFR and VEGFR. (3) PI3K/AKT/mTOR pathway inhibitor: the PI3K/AKT/mTOR pathway plays an important role in tumor cell proliferation, angiogenesis and metastasis, which is activated in breast cancer. Inhibitors of this pathway can inhibit the growth of breast cancer tumors and cause apoptosis of cancer cells, which are expected to become new drugs for TNBC targeted therapy (Costa et al., 2018; Kontzoglou and Garmpis, 2019). Our research shows that XHP can target the PI3K/AKT/mTOR pathway. (4) Silk-threonine protein kinase-related targeted drugs: PIMI kinase is a serine protein kinase and clinical studies have shown that PIMI kinase inhibitors can be used as new targeted therapies for patients with TNBC (Horiuchi et al., 2016). Studies have confirmed that the expression level of PIMI in TNBC tissues are higher than that in normal breast tissue and hormone receptor-positive breast cancer tissues (Zhao et al., 2017). In addition, PIMI plays an important role in the growth and proliferation of TNBC cells expressing MYC (Brasó-Maristany et al., 2016). Our chemical informatics studies show that XHP can regulate serine–threonine protein kinase-mediated biological processes. In the future, XHP and Olibanum may be used as sensitizers, and combined with monoclonal antibodies or inhibitors can significantly increase the objective response rate or survival rate of patients with TNBC.

The second aspect is immunotherapy. Tumor immunotherapy controls and kills tumor cells by stimulating or modulating the immune system to enhance the anti-tumor immunity of the tumor microenvironment (Lee Ventola, 2017). In the future, the most promising immunotherapeutic drug for the treatment of TNBC is the immunological checkpoint inhibitor (ICPI) (Li et al., 2018b). In addition, TNBC has a unique immune microenvironment. TNBC overexpresses VEGF, other molecules that promote tumor cell growth and migration, and has more tumor infiltrating lymphocytes (TIL) and tumor-associated macrophages (TAMs) (Saraiva et al., 2017; Yu and Di, 2017; Romero-Cordoba et al., 2019). The immune microenvironment of tumor cells is a place where the immune escape of tumor cells and the immune surveillance of the human immune system compete against each other. Therefore, the tumor immune microenvironment is closely related to the occurrence, development and prognosis of TNBC. The results of biological processes and signaling pathway enrichment analysis show that XHP can regulate the immune process in TNBC. Li et al. (2018a) and Su et al. (2018) have confirmed that XHP can inhibit tumor cell growth by improving the immunosuppressive state of the tumor microenvironment, promoting apoptosis of Treg cells in tumor microenvironment, and reversing immune escape.

The third aspect is endocrine therapy. Endocrine therapy refers to inhibition of tumor cell growth by regulating hormone levels and effects in the body. However, since the ER and PR in patients with TNBC are negative, traditional endocrine therapy is ineffective for them (Márquez-Garbán et al., 2019). Current research showed that androgen receptor (AR) inhibitors are potential therapeutic agents for TNBC, and AR signaling cascades can be inhibited by multiple pathways (Kono et al., 2017). Epidemiological surveys showed that 10%-35% of patients with TNBC express AR, while AR overexpression usually indicates poor prognosis (Barton et al., 2015). Therefore, blocking the expression of AR becomes a viable endocrine therapy for TNBC. For example, bicalutamide, the anti-AR inhibitor, achieved clinical benefit in 19% of patients in phase III clinical trials (Abe et al., 2014; Venema et al., 2019). In addition, TNBC’s endocrine therapy also includes targets such as gonadotropin-releasing hormone and growth hormone-releasing hormone, but the related studies are few.

### Transcriptome Analysis of *Olibanum*-Treated MD-MB 231 Cells

#### Differentially Expressed Gene of *Olibanum*-Treated MD-MB 231 Cells

**Figure 14:**
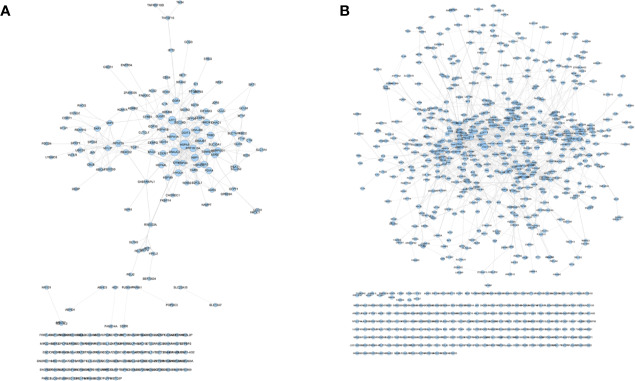
DEGs PPI networks. **(A)** DEGs PPI network of *Olibanum*-treated MD-MB 231 cells; **(B)** DEGs PPI network of β-boswellic acid -treated MD-MB 231 cells.

The transcriptome data comes from GSE102891. When performing the analysis, select “Boswellia Serrata Extract 128 ug/ml” as the experimental group, and select “control” as the control group to obtain gene expression data. Gene with a P value of <0.05 and Log2FC>1 or <−1 is considered to be a differentially expressed gene (DEG) (**Figure S5**). PCA plot showed that the results of *Olibanum* group, β-boswellic acid group and control group were significantly different (**Figure S6A**). The different clustering between Olibanum group and β-boswellic acid group were shown in **Figure S6B**. A total of 227 genes were identified as DEGs and were used to construct DEGs PPI network of *Olibanum*-treated MD-MB 231 cells (**Figure 14A**). The details of each DEG was shown in **Table S5**.

In **Figure 14**, the size of each node is related to its Degree; the bigger nodes have the larger value of Degree. The width of line is associated with its Edge Betweenness; the wider lines have the larger value of Edge Betweenness. In this network, the top 21 targets are: HSPA5 (33 edges), DDIT3 (28 edges), DNAJC3 (25 edges), ATF3 (24 edges), XBP1 (21 edges), HSPA1A (21 edges), PPP1R15A (19 edges), DNAJB9 (18 edges), TRIB3 (18 edges), HYOU1 (18 edges), PDIA3 (18 edges), ASNS (17 edges), RPS27A (16 edges), HMOX1 (16 edges), HERPUD1 (16 edges), DNAJB1 (16 edges), PDIA4 (16 edges), TXNRD1 (16 edges), EGR1 (15 edges), DUSP1 (15 edges), ERN1 (15 edges). These targets are considered to be the core targets of this network.

In the central node of the network, HSPA5, DDIT3, DNAJC3, ATF3, XBP1, PPP1R15A, DNAJB9, TRIB3, PDIA3, ASNS, HERPUD1, DNAJB1, PDIA4, TXNRD1, DUSP1, ERN1 are genes related to endoplasmic reticulum stress, which is essential for regulating the control of protein quality by endoplasmic reticulum and maintaining the balance of redox state (Xu and Park, 2018; Amen et al., 2019; Gao et al., 2019; Kim E. K. et al., 2019). Current research shows that Endoplasmic Reticulum Associated Unfolded Protein Response (UPR) and endoplasmic reticulum stress can affect the migration and invasion characteristics of breast cancer cells. The mechanisms include extracellular matrix (ECM) remodeling, cell adhesion modification, chemoattraction, epithelial-mesenchymal transition (EMT), regulation of signaling pathways associated with cell migration, and cytoskeletal remodeling, which in turn promotes breast cancer cell migration and invasion (Cook et al., 2016; Han and Wan, 2018; Sisinni et al., 2019). Hence, in the future, targeting UPR and breast cancer stress may be potential targeted therapeutic strategies for the treatment of breast cancer.

HSPA5, as a gene related to endoplasmic reticulum stress, plays an important role in the biological processes of breast cancer; inhibition of HSPA5 can inhibit TNBC cell migration and invasion (Chen et al., 2015). The endoplasmic reticulum stress-related gene DDIT3 also participates in the development of TNBC by regulating autophagy and apoptosis of TNBC cells (Singha et al., 2013). *In vitro* and *in vivo* showed that LYN-1604 exerts an anti-TNBC effect by targeting ULK1-regulated autophagic death; its induced autophagic death is closely related to these key genes such as ATF3, RAD21 and caspase3 (Ouyang et al., 2017; Zhang et al., 2017). The heat shock protein HYOU1 (also known as Orp150) plays an important role in hypoxia/ischemia and angiogenesis, which is overexpressed in certain invasive breast cancers, and its overexpression appears to be associated with poor prognostic indicators (Li et al., 2019b). In metastatic breast and ovarian cancer, HSPA1 has a different lysine methylation, and unmethylated HSPA1 shows potential as a prognostic marker in potentially highly serous carcinomas (Jakobsson et al., 2015).

#### Enrichment Analysis of DEGs PPI Network of *Olibanum*-Treated MD-MB 231 Cells

**Figure 15:**
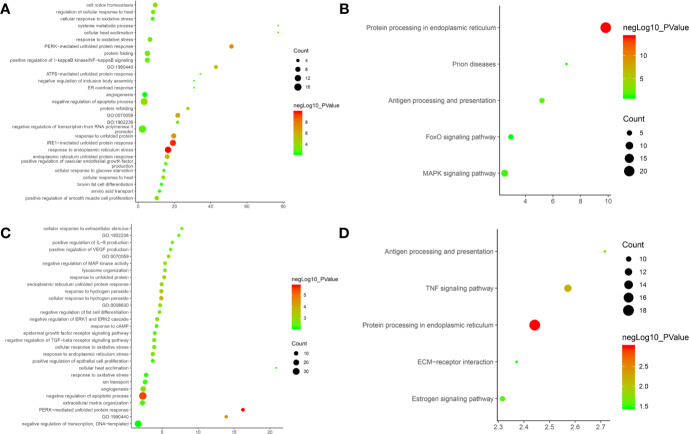
Bubble chart. **(A)** biological processes of *Olibanum*-treated MD-MB 231 cells; **(B)** signaling pathways of *Olibanum*-treated MD-MB 231 cells; **(C)** biological processes of β-boswellic acid -treated MD-MB 231 cells; **(D)** signaling pathways of β-boswellic acid -treated MD-MB 231 cells.

After the enrichment analysis, several biological processes and signaling pathways are obtained. The results of enrichment in the DAVID database indicate that these DEGs are primarily involved in biological processes associated with endoplasmic reticulum stress response, such as cellular response to topologically affected protein, MAPK signaling pathway, FoxO signaling pathway and so on. The main biological processes and signaling pathways were shown in **Figures 15A, B**. The details of each biological processes and signaling pathway were shown in **Table S6**.

Metascape is a portal that provides genetic annotation and analysis resources to help biologists understand one or more gene lists (http://metascape.org/gp/index.html#/main/step1) (Zhou et al., 2019). Metascape’s enrichment analysis of DEGs adds new biological processes and signaling pathways; compared to DAVID, the Reactom pathway is added, and the background annotation genes are more complete (**Figure S7**). Interestingly, the results of pathway enrichments indicate that Ferroptosis has the highest enrichment, and current studies show that induction of Ferroptosis has become an important strategy for the treatment of tumors (Galluzzi et al., 2018); especially in breast cancer, ferroptosis induces tumor cell death by activating the -ATF4 pathway of GCN2-eIF2α in TNBC cells (Chen et al., 2017). This suggests that the *Olibanum* extract may contain components that potentially induce Ferroptosis. The details of the biological processes, signaling pathways and Reactome pathways were shown in **Table S7**.

### Transcriptome Analysis of β-Boswellic Acid-Treated MD-MB 231 Cells

#### Differentially Expressed Gene of β-Boswellic Acid-Treated MD-MB 231 Cells

The transcriptome data comes from GSE102891. When performing the analysis, select “3-O-Acetyl-β-boswellic acid 46 ug/ml” as the experimental group, and select “control” as the control group to obtain gene expression data. Gene with a P value of <0.05 and Log2FC >1 or <−1 is considered to be a differentially expressed gene (DEG) (**Figure S8**). A total of 950 genes were identified as DEGs and were used to construct DEGs PPI network of β-boswellic acid -treated MD-MB 231 cells (**Figure 14**). In **Figure 14B**, the size of each node is related to its Degree; the bigger nodes have the larger value of Degree. The width of line is associated with its Edge Betweenness; the wider lines have the larger value of Edge Betweenness. In this network, the top 20 targets are: FOS (51 edges), CXCL8 (49 edges), CD44 (46 edges), HSPA5 (39 edges), SIRT1 (38 edges), PTGS2 (37 edges), HDAC3 (36 edges), TIMP1 (33 edges), H2AFX (30 edges), TGFB1 (29 edges), UBE2C (28 edges), EGR1 (28 edges), ATF3 (27 edges), SERPINE1 (27 edges), HIST2H2BE (27 edges), CEBPB (27 edges), STUB1 (26 edges), TUFM (26 edges), CLPP (25 edges), HSPA1A (25 edges). Compared with DEGs PPI network of *Olibanum*-treated MD-MB 231 cell, their common targets are: HSPA5, ATF3, HSPA1A, EGR1. The details of each biological processes and signaling pathway were shown in **Table S8**.

FOS, HSPA5, SIRT1, PTGS2, HDAC3, TIMP1, TGFB1, ATF3, CEBPB, CLPP are involved in endoplasmic reticulum-related biological processes such as endoplasmic reticulum stress response and endoplasmic reticulum unfolded protein response (Wilkinson, 2019; Limia et al., 2019; Omidkhoda et al., 2019), which mediate the development of breast cancer. Recent studies have shown that the cell surface adhesion receptor CD44 is a key positive regulator of PD-L1 expression in TNBC and non-small cell lung cancer (NSCLC) (Kong et al., 2019). The Notch-mediated tumor-interstitial-inflammatory network promotes TNBC invasiveness and CXCL8 expression (Liubomirski et al., 2019). High expression of UBE2C is a potential factor for poor prognosis of TNBC. Furthermore, loss of BRCA1 function results in increased expression of UBE2C and chemoresistance to doxorubicin in breast cancer cells (Qin et al., 2017). The up-regulation of Serpine2 promotes breast cancer cell metastasis and reduces patient survival (Jin et al., 2017). Meanwhile, recent studies have shown that CHIP/STUB1 ubiquitin ligase is a negative chaperone molecule of HSP90/HSC70, which is reduced or lost in breast cancer. The absence of CHIP reshapes the cellular transcriptome and releases key cancer-promoting factors, such as the matrix degrading enzymes of the cathepsin family (Luan et al., 2018). In addition, downregulation of TUFM can induce epithelial-mesenchymal transition, and analogs of resveratrol HS-1793 can down-regulate its expression and increase anticancer activity against MCF-7 cells (Jeong et al., 2012).

#### Enrichment Analysis of DEGs PPI Network of β-Boswellic Acid-Treated MD-MB 231 Cells

After the enrichment analysis, several biological processes and signaling pathways are obtained. The results of enrichment in the DAVID database indicate that these DEGs are primarily involved in biological processes associated with endoplasmic reticulum stress response, such as PERK-mediated unfolded protein response, negative regulation of apoptotic process, and so on. The signaling pathways are protein processing in endoplasmic reticulum, TNF signaling pathway, Antigen processing and presentation, Estrogen signaling pathway, ECM-receptor interaction. The main biological processes and signaling pathways were shown in **Figures 15C, D**.

The biological processes in the Metascape database are similar to those in the DAVID database, in a different order (**Figure S9**). The details of the biological processes, signaling pathways and Reactome pathways were shown in **Table S9**.

In summary, through further analysis of transcriptomics data, it is found that the TNBC-related biological processes regulated by *Olibanum* extracts and β-boswellic acid mainly include endoplasmic reticulum stress response, oxidative stress, angiogenesis, inflammatory response, cell migration and adhesion, hypoxia induction, and autophagy. Of particular importance, the endoplasmic reticulum stress response is thought to be the primary upstream mechanism by which *Olibanum* extracts and β-boswellic acid play a role in TNBC cells. Hence, endoplasmic reticulum stress is a potential target in cancer therapy due to its important role in cancer development.

## Conclusion

XHP may exert anti-TNBC effects through regulating biological processes, signaling pathways, targets found in this study. Meanwhile, the ability of *Olibanum* extracts and β-boswellic acid to induce endoplasmic reticulum stress and subsequently activate tumor cell death programs confirms that they are a promising class of anticancer agents. In addition, the *Olibanum* extracts may be used as an inducer of TNBC’s Ferroptosis in the future.

## Data Availability Statement

All datasets for this study are included in the article/**Supplementary Material**.

## Author Contributions

LZ and KY dominated the concept and carried out a comprehensive design. LL, XX, AG, and TB were participants in the concept and design. KY, LZ, AG, TB, and LL are responsible for data analysis and interpretation in the chemical informatics section. TX and XX, and LL are responsible for data analysis and interpretation in experiments. KY, LZ, AG, and TB drafted the paper. LL and XX supervised the study. All authors participated in the analysis and interpretation of data and approved the final paper. KY, LZ, AG and TB should be considered joint first author. LL is the first corresponding author because she supervised the study.

## Funding

This research is supported by Scientific Research Project of Hunan Provincial Department of Education (No. 19B434) and Double First-Class University Project of Hunan Province (Xiangjiaotong [2018]469).

## Conflict of Interest

The authors declare that the research was conducted in the absence of any commercial or financial relationships that could be construed as a potential conflict of interest.
