# Supplementary material for: Exploring the Regulation Mechanism of Xihuang Pill, Olibanum and β-Boswellic Acid on the Biomolecular Network of Triple-Negative Breast Cancer Based on Transcriptomics and Chemical Informatics Methodology
Source: Front Pharmacol. 2020 Jun 11;11:825. doi: 10.3389/fphar.2020.00825 (PMC7300251; doi:10.3389/fphar.2020.00825)
Supplement: Supplementary file 1 [file DataSheet_1.zip › Supplementary Figures.DOCX]

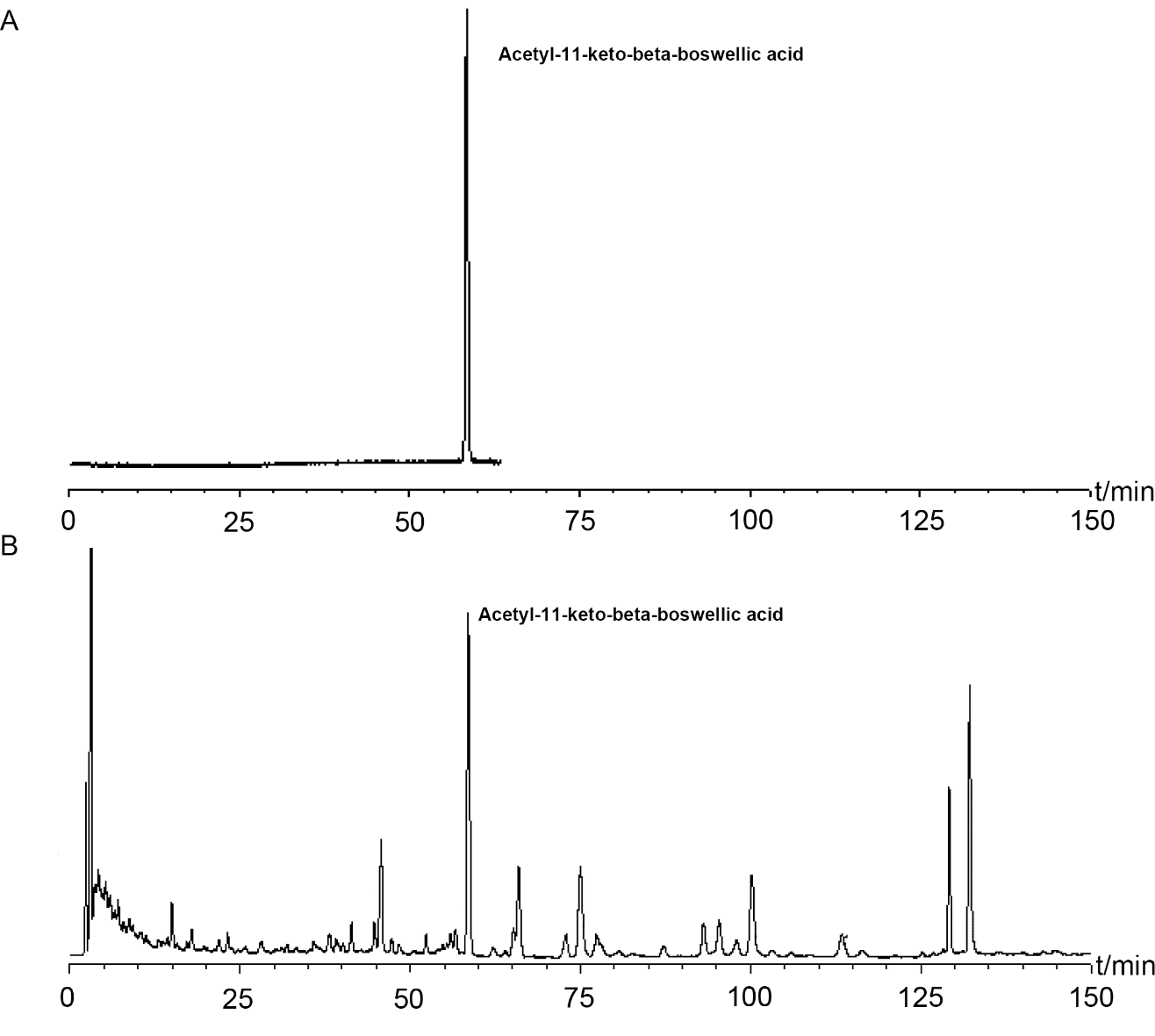


Figure S1 XHP’s fingerprint (A: acetyl-11-keto-β-boswellic acid reference substance; B XHP sample)


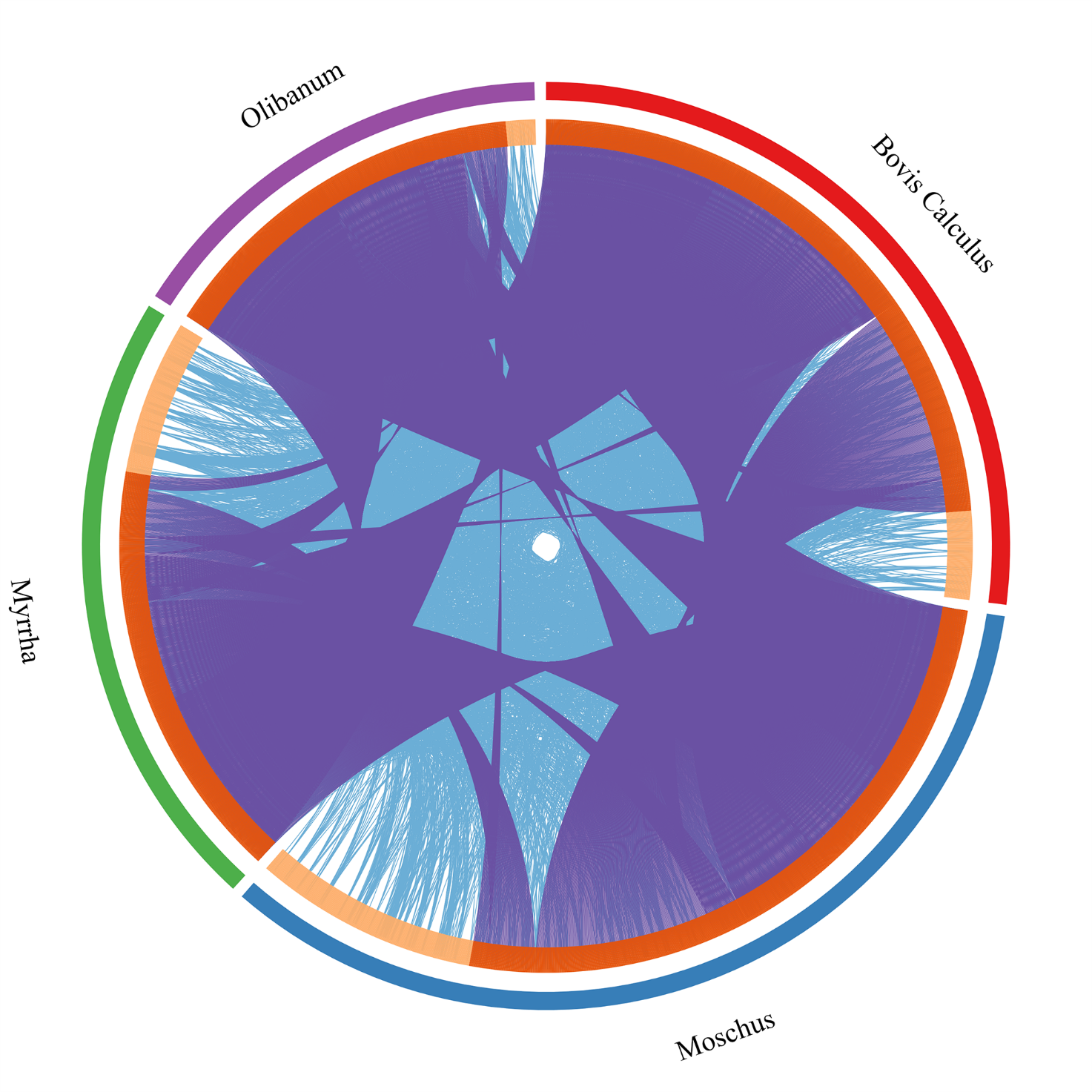


Figure S2 The circles plot of potential targets distribution


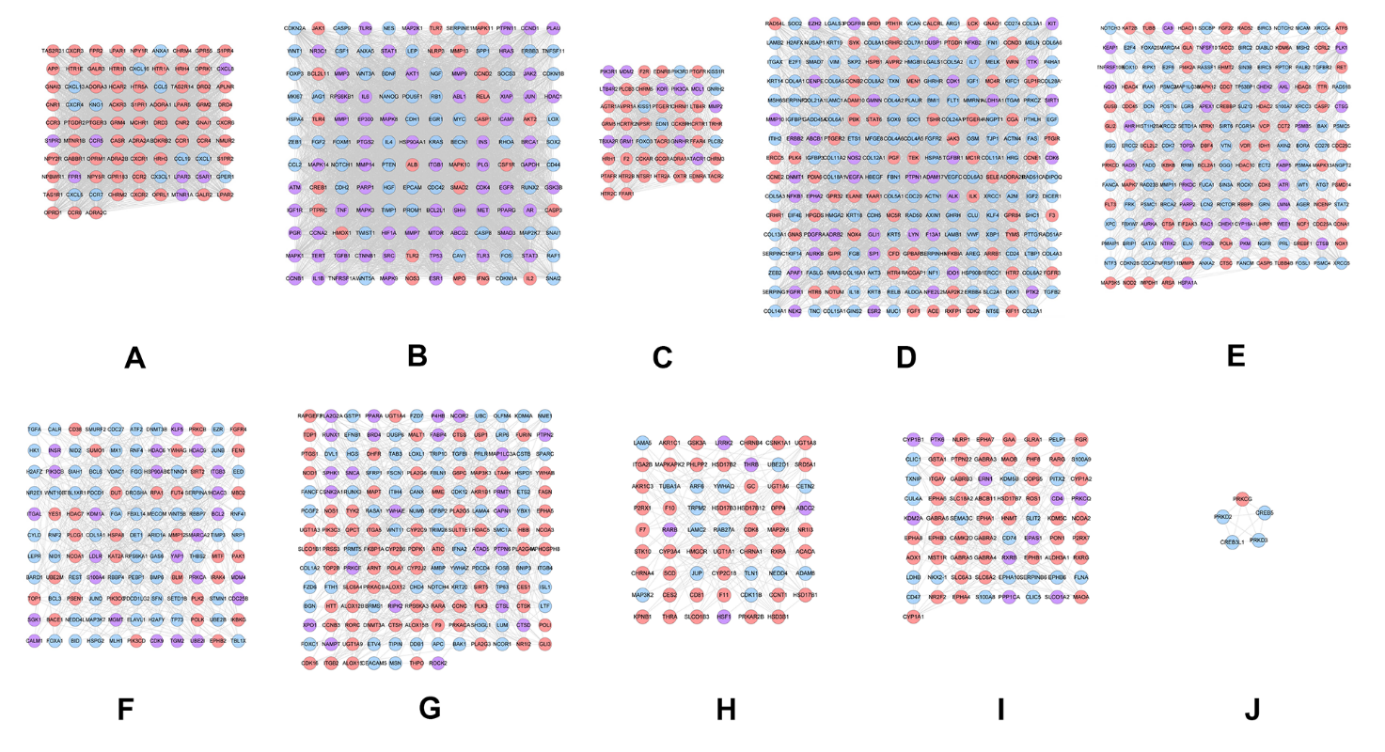


Figure S3 Cluster of XHP-TNBC PPI network (A, B, C, … stand for cluster 1, 2, 3, …; Purple circle stands for XHP-TNBC; Blue circle stands for TNBC genes; Pink circle stands for XHP targets)


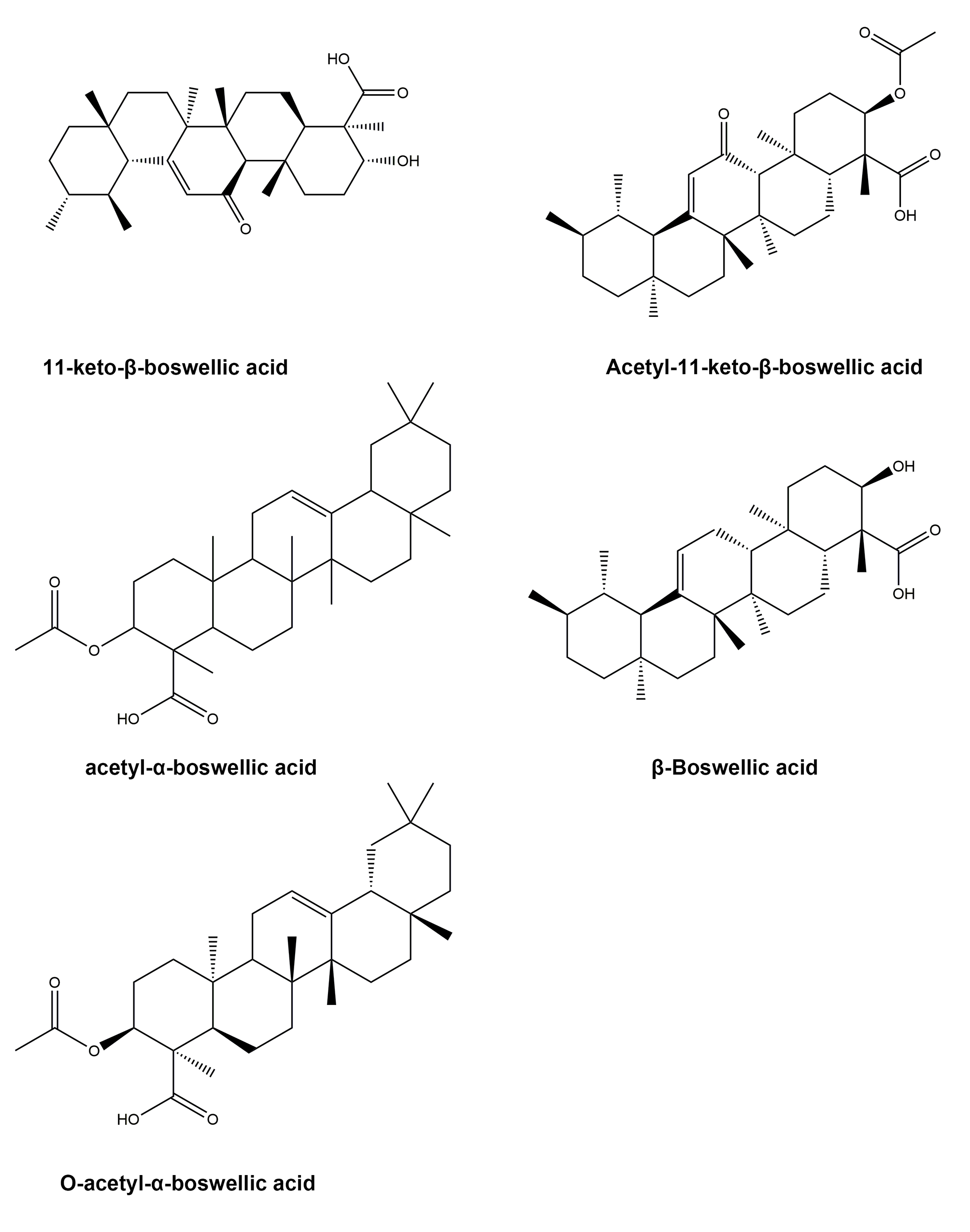


Figure S4 The main anti-cancer compounds of *Olibanum.*


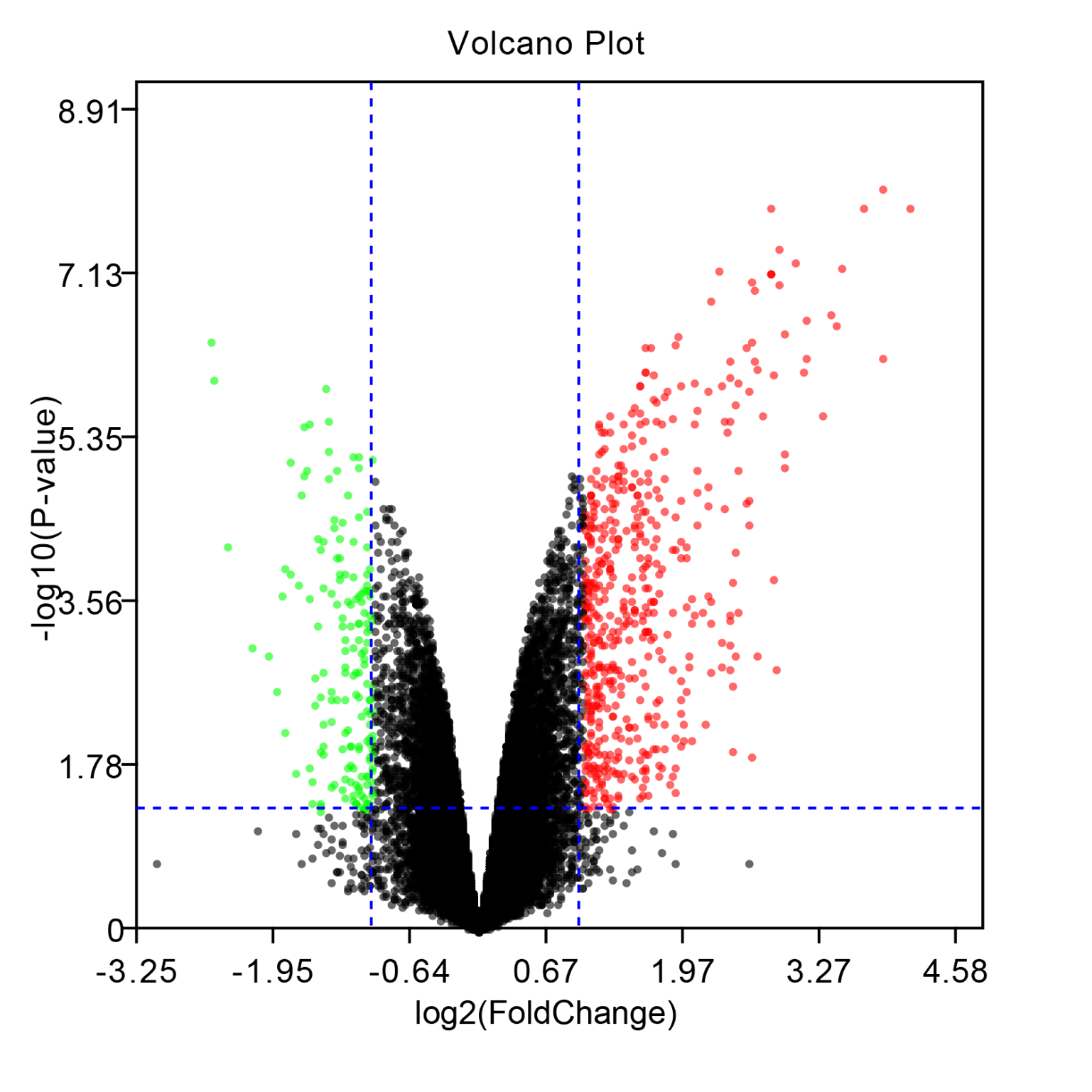


Figure S5 Volcano Plot (Red point stands for up-regulated gene; Green point stands for down-regulated gene. Black point stands for gene that is not differentially expressed)


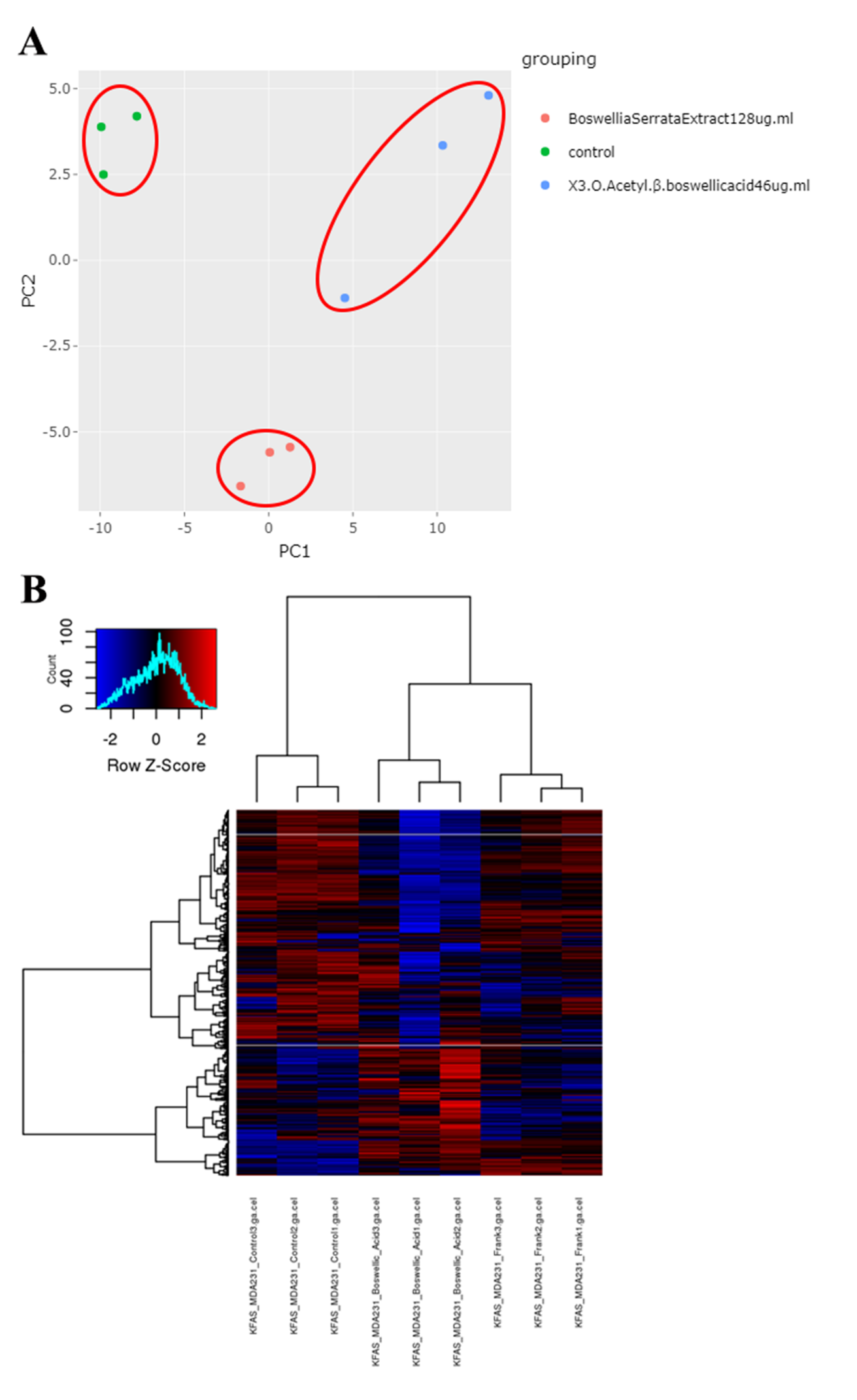


Figure S6 Quality control of the GSE102891 dataset (A: PAC plot; B: clustering heatmap)


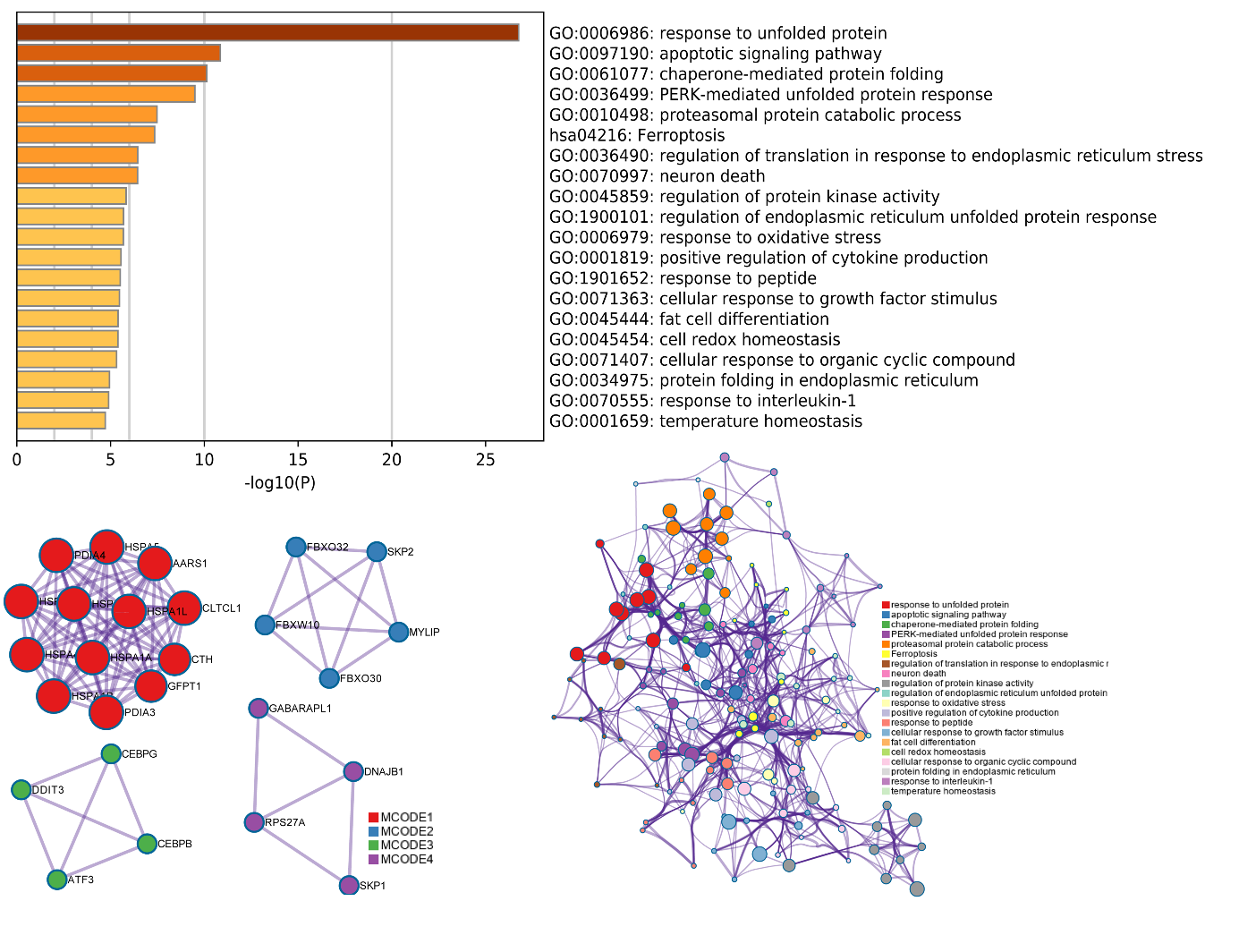


Figure S7 The results of Metascape's enrichment analysis of DEGs of *Olibanum*-treated MD-MB 231 cells


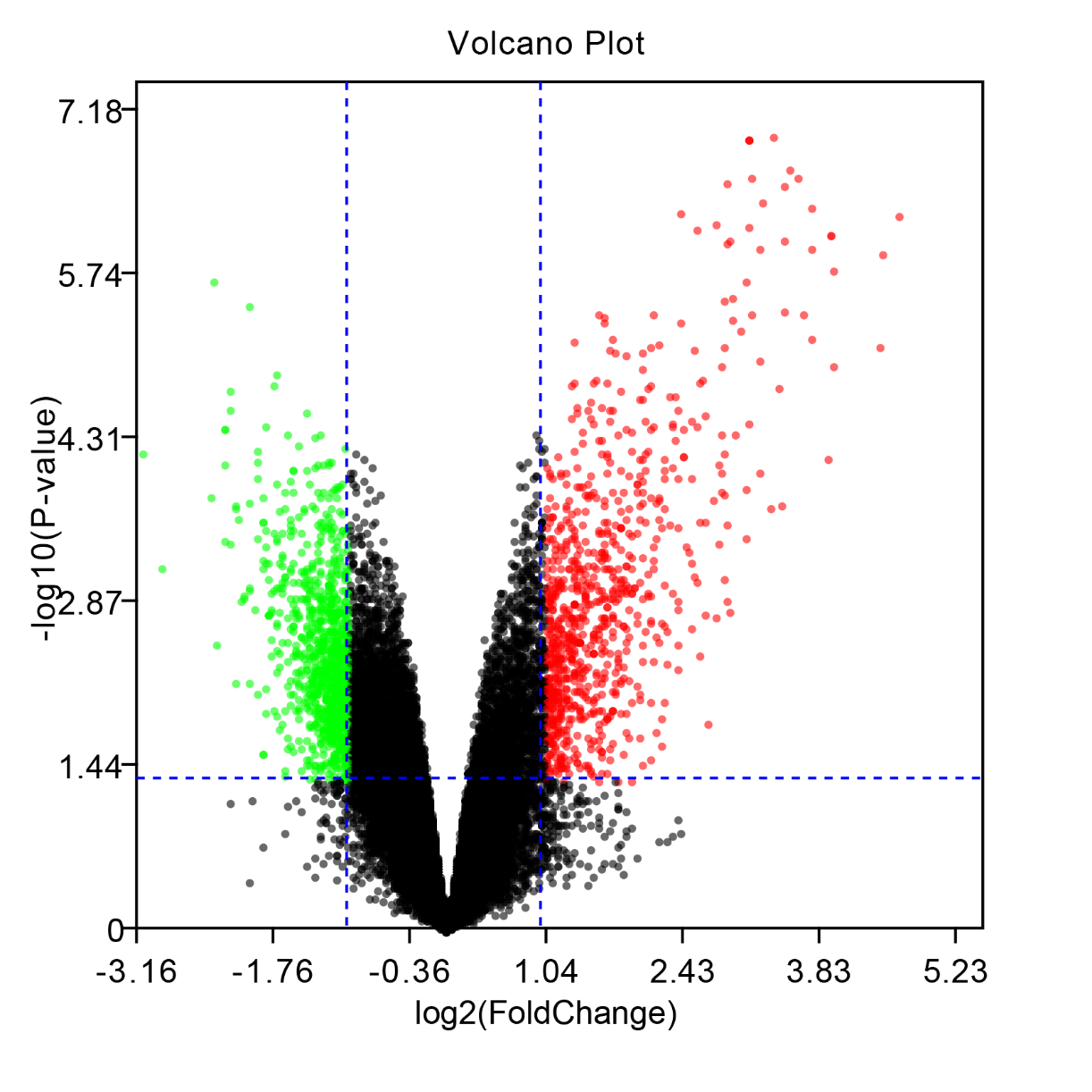


Figure S8 Volcano Plot (Red point stands for up-regulated gene; Green point stands for down-regulated gene. Black point stands for gene that is not differentially expressed)


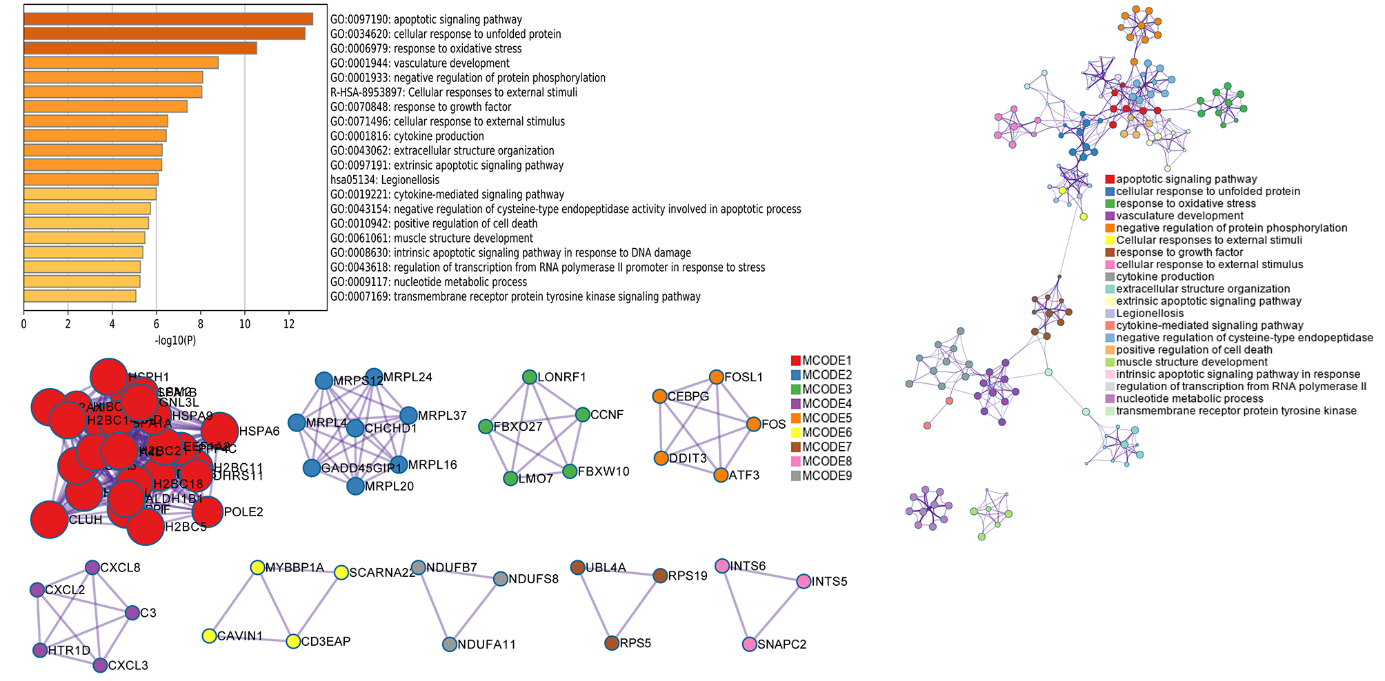


Figure S9 The results of Metascape's enrichment analysis of DEGs of β-boswellic acid -treated MD-MB 231 cells
